# Diffusion‐Optimized Long Lifespan 4.6 V LiCoO_2_: Homogenizing Cycled Bulk‐To‐Surface Li Concentration with Reduced Structure Stress

**DOI:** 10.1002/advs.202308258

**Published:** 2024-01-30

**Authors:** Kang Wu, Peilin Ran, Baotian Wang, Fangwei Wang, Jinkui Zhao, Enyue Zhao

**Affiliations:** ^1^ Songshan Lake Materials Laboratory Dongguan 523808 P. R. China; ^2^ Beijing National Laboratory for Condensed Matter Physics Institute of Physics Chinese Academy of Sciences Beijing 100190 P. R. China; ^3^ Institute of High Energy Physics Chinese Academy of Sciences Beijing 100049 P. R. China; ^4^ Spallation Neutron Source Science Center Dongguan Guangdong 523803 P. R. China

**Keywords:** homogenized lithium‐ion concentration, lithium‐ion battery, long cycle life, reduced structural stress

## Abstract

Increasing the charging cut‐off voltage (e.g., 4.6 V) to extract more Li ions are pushing the LiCoO_2_ (LCO) cathode to achieve a higher energy density. However, an inhomogeneous cycled bulk‐to‐surface Li distribution, which is closely associated with the enhanced extracted Li ions, is usually ignored, and severely restricts the design of long lifespan high voltage LCO. Here, a strategy by constructing an artificial solid–solid Li diffusion environment on LCO's surface is proposed to achieve a homogeneous bulk‐to‐surface Li distribution upon cycling. The diffusion optimized LCO not only shows a highly reversible capacity of 212 mA h g^−1^ but also an ultrahigh capacity retention of 80% over 600 cycles at 4.6 V. Combined in situ X‐ray diffraction measurements and stress‐evolution simulation analysis, it is revealed that the superior 4.6 V long‐cycled stability is ascribed to a reduced structure stress leaded by the homogeneous bulk‐to‐surface Li diffusion. This work broadens approaches for the design of highly stable layered oxide cathodes with low ion‐storage structure stress.

## Graphical abstract

The inhomogeneous cycled bulk‐to‐surface Li distribution severely restricts the design of long lifespan high voltage LiCoO_2_ (LCO). By constructing an artificial solid–solid Li diffusion environment on LCO's surface to achieve a homogeneous bulk‐to‐surface Li distribution upon cycling, which suppress the large Li^+^ concentration difference of LCO, as well as the phase transition on the surface, further reducing near surface structural stress.

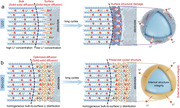

## 1 Introduction

Although various cathode materials have been commercialized for Li‐ion batteries over the past decades, LiCoO_2_ (LCO) is still a strongly competitive cathode for 3 C electronics because of its high volumetric energy density enabled by the ultrahigh tap density (≈4.2 g cm^−3^).^[^ ^1^ ^]^ However, the practical capacity of currently commercialized LCO (170 mA h g^−1^, Li_1−x_CoO_2_, *x* = 0.62; 4.45 V vs. Li/Li^+^) is far from its theoretical one (274 mA h g^−1^), which largely limited the attainable volumetric energy density.^[^ ^2^ ^]^ To further enhance the energy density of LCO, a high charging cut‐off voltage has been employed to increase the amounts of de‐intercalated Li ions.^[^ ^3^ ^]^ For instance, a reversible capacity of >200 mAh g^−1^ (Li_1−x_CoO_2_, *x* > 0.73) for the 4.6 V (vs. Li/Li^+^) charging voltage has been widely reported. The enhanced de‐intercalation amounts of Li ions, unfortunately, can lead to a large structure stress and is not favorable for LCO's lattice structure stability.^[^ ^4^ ^]^ The dissolution of Co, lattice oxygen loss and typical phase transitions (O3 to H1‐3) at the highly delithiated state usually occur, and which can seriously restrict the long‐cycled capacity retention.^[^ ^5^ ^]^ Numerous strategies, including constructing surface protective layer for the elimination of Co dissolution, tuning Co/O electronic band structure for the reduction of lattice oxygen loss and multiple elements doping for the suppression of phase transitions, have been demonstrated effective in stabilizing the high‐delithiated‐state structure of LCO.^[^ ^6^ ^]^ However, till now, rare studies report a long lifespan LCO (e.g., exceed 600 cycles) with a good capacity retention (e.g., >80%), which means that there are still fundamental structure‐stability problems need to be addressed.

With this question in mind, we revisit the Li‐storage structure of LCO. It should be noted that the structure stress cannot only be caused by the large amounts of de‐intercalated Li ions but also be induced by an uneven concentration distribution of Li ions during the Li de‐intercalation process.^[^ ^7^ ^]^ Specifically, there are two kinds of ionic diffusion environments for the Li de‐intercalation: one is solid–solid diffusion that occurs in the bulk structure, the other is solid–liquid diffusion which occurs on the surface contacted with the electrolyte. Compared with the solid–liquid diffusion, the solid–solid one is much slower. Such different diffusion can result in an inhomogeneous bulk‐to surface Li distribution, leading to a large structure stress at the near surface (where shows the largest Li concentration difference). The diffusion‐induced structure stress can cause strains accumulation upon cycling and directly destabilize the Li‐storage structure of LCO.^[^ ^4a,c^ ^]^ Thus, addressing the cycled inhomogeneous bulk‐to‐surface Li distribution is essential for the development of long lifespan LCO.

Herein, to achieve uniform Li^+^ diffusion in the bulk and surface of LCO, we have constructed an artificial solid–solid Li diffusion environment (Li^+^ diffusion coefficient of Li_1.5_Ga_0.5_Ti_1.5_(PO_4_)_3_ (LGTP) is 7.4 x 10^−13^ cm^2^ s^−1^) on the surface of LCO that is the same as the Li^+^ diffusion environment inside the bulk (Li^+^ diffusion coefficient of LCO is 2.7 x 10^−12^ cm^2^ s^–1^), which is equivalent to the first diffusion of Li^+^ to LGTP and then to the liquid electrolyte when Li^+^ are removed from the surface of LCO. In this work, we select the typical Li‐ion conductor LGTP as the research model and highlight the role of cycled Li distribution and diffusion‐induced stress on the Li‐storage structure stability. The slow solid–solid diffusion of Li^+^ from the surface of LCO to LGTP can achieve a homogeneous bulk‐to‐surface Li distribution upon cycling, which suppress the large Li^+^ concentration difference of LCO, as well as the phase transition from layered to rock salt phase on the surface, further reducing near surface structural stress. The diffusion‐optimized LCO (DO‐LCO) shows a record long‐cycle stability with a capacity retention of 80% over 600 cycles at 4.6 V. As directly revealed by in situ X‐ray diffraction stress analysis, DO‐LCO possesses an obviously reduced structure stress upon Li de/‐intercalation process, which is also supported by further stress‐evolution simulation. The long‐cycled Li‐storage structure stability at 4.6 V strongly rely on the reduced structure stress. More broadly, this work highlights the importance of tuning Li diffusion environments in designing highly stable ion‐storage lattice structure.

## 2 Results and Discussion

**Figure 1:**
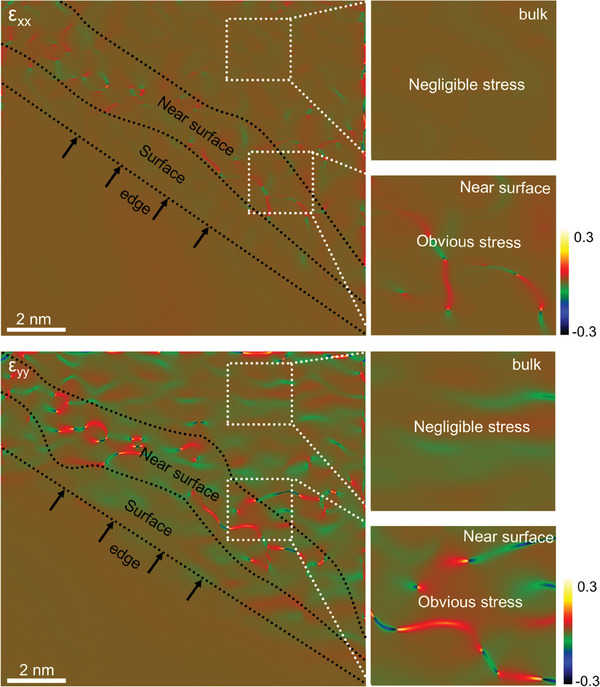
GPA strain map *ε*_xx_ (top panel) and *ε*_yy_ (bottom panel) obtained from HAADF‐STEM data (Figure S1, Supporting Information) of the 4.6 V‐charged LCO sample.

To present a direct evidence for the propose that the inhomogeneous bulk‐to‐surface Li distribution can induce large structure stress, we conducted high angle annular dark field scanning transmission electron microscope (HAADF‐STEM) measurements and geometric phase analysis (GPA) for the 4.6 V‐charged LCO electrode. Theoretically, due to the different Li diffusion velocity between bulk (solid–solid diffusion environment) and surface (solid–liquid diffusion environment), the uneven Li concentration distribution is most obvious at near‐surface area. As predicted, an obvious structure stress is observed at the near‐surface area in both *ε*_xx_ and *ε*_yy_ directions (**Figure** **1**). In contrast, the structure stress at bulk and surface area is relatively lower. The large structure stress located at the near‐surface area can destabilize the layered structure framework, triggering the possible phase transitions and lattice collapse.^[^ ^8^ ^]^ To reduce the structure stress caused by uneven Li^+^ diffusion, a buffered Li^+^ diffusion layer on the surface of LCO were constructed to optimize Li^+^ diffusion environment (design details of DO‐LCO are illustrated in Supporting Information).

**Figure 2:**
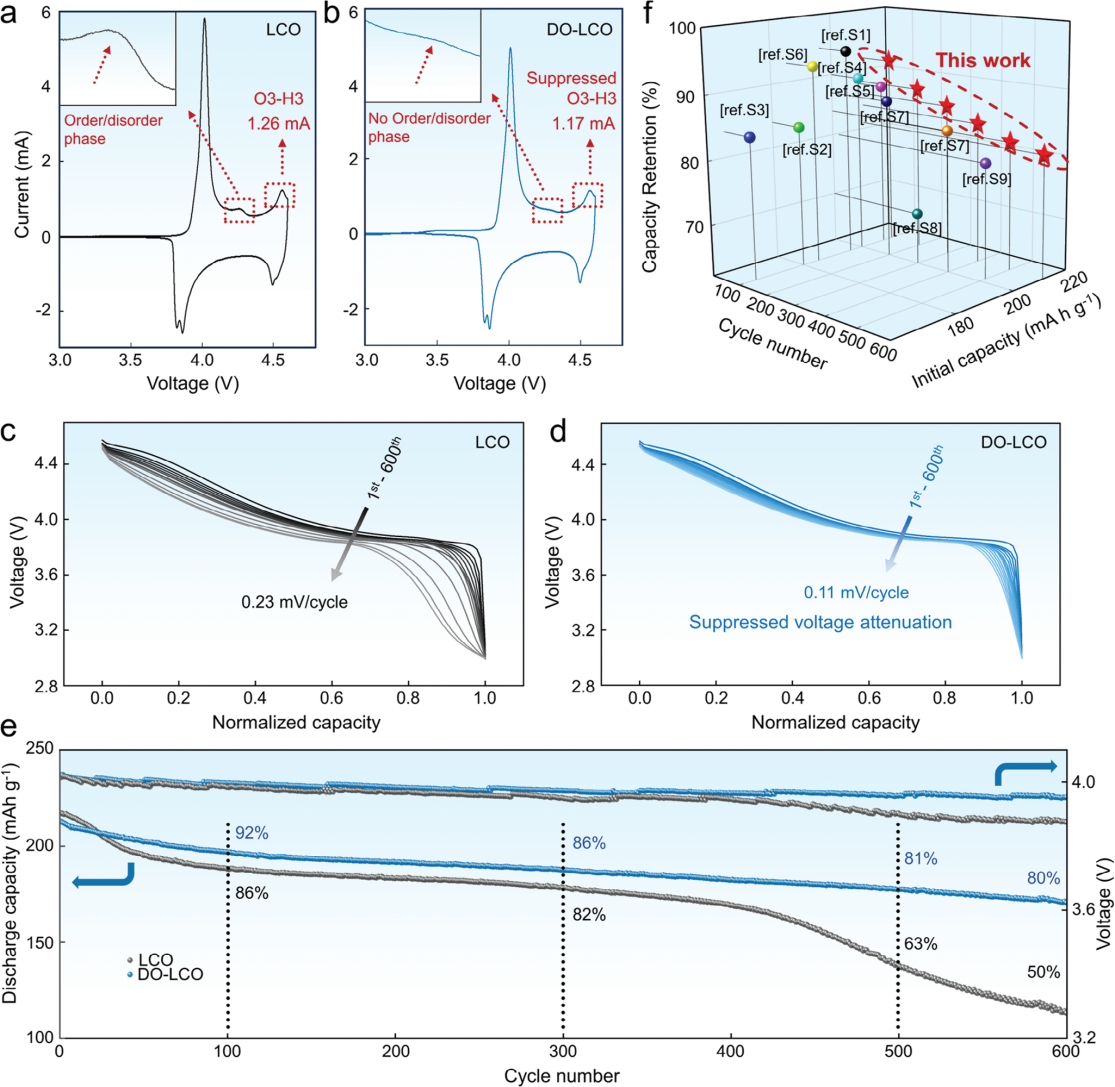
Electrochemical performance. The initial CV curve: a) LCO; b) DO‐LCO, the 2–4 cycled CV curves are shown in Figure S17 (Supporting Information). The variation of discharge curves upon cycling at 1 C: c) LCO; d) DO‐LCO cathodes. e) The long cycle performance and average voltage variation for LCO and DO‐LCO cathodes at 1 C over 600 cycles. f) Comparison of electrochemical performance with other studies.

The cyclic voltammetry (CV) measurements were first conducted to evaluate the effects of optimized Li diffusion on the electrochemical reactions. **Figure** **2**a,b shows the initial CV curve of LCO and DO‐LCO electrodes, respectively. The material undergoes an ordered–disordered transition from O3 phase to monoclinic phase when charged to 4.2 V (50% of Li^+^ are removed). When charged above 4.5 V, LCO undergoes another phase transition from O3 to H1‐3 or O6 phase.^[^ ^9^ ^]^ The order–disorder transition greatly reduces the diffusion coefficient of Li^+^. The H1‐3 phase transition leads to slip on the surface of the O–Co–O layered structure, accompanied by Li^+^ rearrangement, *c*‐axis volume contraction, O loss, and Co dissolution, leading to local surface structure collapse, particle fracture and a sharp decrease in capacity.^[^ ^10^ ^]^ Hence, through constructing an artificial solid–solid Li diffusion environment on LCO's surface, a homogeneous bulk‐to‐surface Li distribution upon cycling can be achieved, which effectively reduce the bulk structure stress and have a positive impact on the bulk‐structure stability. In particular, a characteristic peak of ordered/disordered phase transition appeared in LCO near 4.2 V, but it was not observed in DO‐LCO material. In addition, the reduction of the peak of DO‐LCO material near 4.55 V further indicating that diffusion optimization promotes the homogenization of Li^+^ concentration and effectively inhibits the occurrence of phase transformation. Notably, during the following cycles, the oxidation peak of DO‐LCO electrode always keeps a higher intensity and good reversibility compared with that of LCO electrode (Figure S17a,b, Supporting Information). This observation indicates that DO‐LCO electrode shows more reversible redox processes. In the meanwhile, as depicted in Figure 2c–e, the average voltage attenuation of DO‐LCO cathode is only 0.017% (0.11 mV per cycle) which is much lower than LCO of 0.035% (0.23 mV per cycle), demonstrating that homogenized Li^+^ concentration also has a positive effect on the suppression of voltage attenuation. More importantly, a long lifespan of DO‐LCO (exceed 600 cycles) with a good capacity retention (>80%) was achieved (Figure 2e). The cycle stability was verified at a high rate of 10 C after activation at the rate of 0.2 C for 1 cycle and the results are shown in Figure S20 (Supporting Information). Significantly, the discharge capacity of DO‐LCO delivered 160 mA h g^−1^ with 92% capacity retention at 10 C after 100 cycles, and even a capacity of up to 130 mA h g^−1^ with a capacity retention rate of 74% after 1000 cycles. In addition, galvanostatic intermittent titration technique (GITT) was used to explore the kinetic process of Li^+^ diffusion and the evolution of polarization of LCO and DO‐LCO during charging and discharging (Figure S21, Supporting Information).^[^ ^11^ ^]^ The low electrochemical polarization and higher Li^+^ diffusion coefficient for DO‐LCO indicates that the optimized Li^+^ diffusion is also favorable for the Li^+^ de‐intercalation.^[^ ^12^ ^]^ What's more, our work was compared with previously published studies on capacity retention and initial discharge capacity of different cycles. It is shown that DO‐LCO cathode shows a record long‐cycled stability and high initial reversible capacity (Figure 2f; Table S7, Supporting Information). The excellent electrochemical performance of diffusion optimized material indicates that homogeneous bulk‐to‐surface Li distribution is essential for the development of long lifespan LCO.

**Figure 3:**
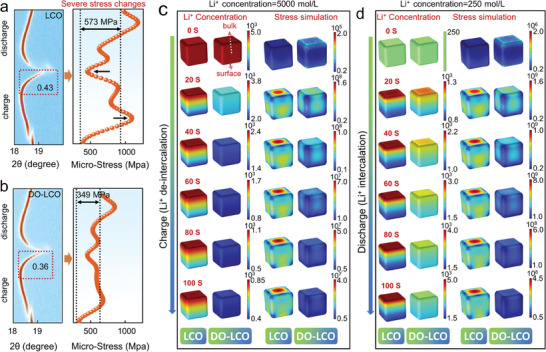
Structure/stress evolutions and simulation Li^+^ concentration/stress distribution. The selected in situ XRD (003) peak and the refined lattice strain change for the a) LCO and ( b) DO‐LCO electrodes, see also Figures S22,S23 (Supporting Information) for more detailed in situ XRD spectra. c,d) The simulation diagram of Li^+^ concentration distribution and stress simulation diagram for LCO and DO‐LCO at the bulk and surface with COMSOL software at charge and discharge.

The in situ X‐ray diffraction (XRD) measurement was applied to investigate structural evolutions for LCO and DO‐LCO cathodes. When the material is charged to 4.3 V, the (003) diffraction peak gradually moves to a lower angle, corresponding to the order–disorder transition from O3 phase to monoclinic structure and the increase of cell parameters (**Figure** **3**a).^[^ ^13^ ^]^ After a further de‐intercalation of Li^+^, another transition from O3 phase to H1–H3 or O6 phase leads to a serious contraction of the *c* lattice parameters, resulting in the (003) peak moving to a higher angle.^[^ ^14^ ^]^ The order–disorder transition will greatly reduce the diffusion coefficient of Li^+^ and the transformation from O3 phase to H1–H3 or O6 phase will lead to large mechanical stress of LCO. In addition, we have obtained the angle and peak intensities corresponding to the H1–3 phase of two materials in Figure 3b; Table S8 (Supporting Information). It is worth noting that the peak intensities and shifting of LCO corresponding to the H1–3 phase at highly delithiated state are higher than that of DO‐LCO material, indicating that diffusion optimization promotes the homogenization of Li^+^ concentration and effectively inhibits the occurrence of phase transformation. Specially, the maximum change in cell parameter *a* during charging process decreased from 2.1% of LCO to 0.3% of DO‐LCO (Figures S22,S23, Supporting Information). Stress generated by O3‐H1‐3 phase transformation causes microcracks in the material and gradually extends to the surface, which promotes electrolyte penetration. The propagation of microcracks causes more surfaces of the material to be exposed to the electrolyte and accelerates the structural degradation of the material. The structure stress evolution during the charging and discharging process was further obtained through refinement analysis (right panel of Figure 3a,b). The stress change for DO‐LCO (379 MPa) during the process of charging and discharging was significantly smaller than that of LCO (573 MPa), indicating that a uniform Li^+^ concentration can reduce the severe structural stress accumulation of LCO and effectively enhance the stability of the crystal structure.^[^ ^4a–c^ ^]^

In order to further elucidate the coupling relationship between Li^+^ concentration and structural stress during highly delithiated state, COMSOL software was used to simulate the Li^+^ concentration and structural stress distribution from the bulk to the surface of the material. The size of the particle model is similar to the actual particle observed by scanning electron microscopy (SEM), with a diameter of 4 microns and a height of 4 microns and a comparison sample of modified particles covered with solid electrolyte (0.1 µm). The initial Li^+^ concentration of LCO and DO‐LCO materials in charge and discharge state is consistent with the electrochemical data. It is assumed that the initial Li^+^ concentration of LCO and DO‐LCO material is 5000 mol L^−1^. For all uncharged samples, the Li^+^ concentration distribution on the material bulk and surface is relatively uniform, corresponding to the red scale on the right (0 s). With the process of de‐intercalation (0 s→100 s), the fast diffusion of Li^+^ in the electrolyte leads to the low concentration of Li^+^ on the surface of LCO. While the Li^+^ migration from the bulk to the surface show sluggish Li^+^ transfer kinetic, which also leads to the phenomenon that the Li^+^ concentration of the inner bulk is higher than that at the surface, forming an obvious uneven distribution of concentration, especially in the state of full charge (Li^+^ de‐intercalation reaches 100 s) (left panel of Figure 3c). In addition, the concentration distribution of Li^+^ in the materials during the insertion process is further studied, assuming that the initial Li^+^ concentration of LCO and DO‐LCO is 250 mol L^−1^. As the concentration of Li^+^ increases, LCO material still exhibit concentration gradients on the bulk and surface (left panel of Figure 3d). In contrast, unlike the solid–liquid interface formed between LCO surface and electrolyte, the formation of a solid–solid interface with a unique Li^+^ diffusion environment on LCO surface can significantly optimize the diffusion of Li^+^. Acts as solid–solid interface layer for Li^+^ diffusion between LCO surface and the electrolyte, the more uniform Li^+^ concentration distribution and smaller concentration gradient are achieved during the whole lithiation and de‐lithiation process from 0 s to 100 s (left panel of Figure 3c,d). In addition, the Li^+^ concentration during the 100th charge and discharge process was further verified. Compared with the initial charge, the initial Li^+^ concentration of LCO material is defined as 4290 mol L^−1^ at the beginning of the 100th charge due to the capacity loss, and the Li^+^ concentration at the beginning of the 100th discharge is defined as 0 mol L^−1^ (charging and discharging efficiency has almost reached 100%). It can be observed that the interior and surface of LCO material also show a similar Li^+^ concentration gradient (the left panel of Figure S15a,b, Supporting Information).

With the de‐intercalation of Li^+^ during the charging process, the electrostatic repulsion between the oxygen layers and the stress in the material increases, causing the electrode to expand along the *c*‐axis. Meanwhile, the uneven concentration of Li^+^ also leads to the accumulation of micro‐stresses and micro‐strains within the grains, exposing more surface of the material to the electrolyte and accelerating the structural degradation of the material. The severe Li^+^ concentration gradient in LCO material cause the corresponding strain difference, which presents different stress characteristics compared to the pristine state before cycling (right panel of Figure 3c). As further discharge proceeds (Li^+^ insertion), the equivalent local stress continues to increase, much higher than the equivalent stress before cycling (right panel of Figure 3c). In fact, such a large and uneven stress is sufficient to cause adverse and irreversible structural damage to LCO electrode after 100 cycles. However, DO‐LCO material shows a smaller structure stress value and a higher reversibility in the initial cycle, and the stress distribution is still relatively uniform even after 100 cycles (right panel of Figure 3c,d; Figure S24a,b, Supporting Information). It is definitely concluded that the structure stress of DO‐LCO hardly induces severe damage to the layered structure, which is totally different from the pristine LCO sample.

**Figure 4:**
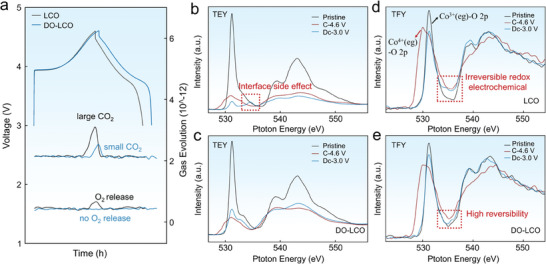
a) *Operando* DEMS results of the initial charge/discharge processes for LCO and DO‐LCO. Ex situ sXAS results for the O K‐edge obtained in TEY mode for the b) LCO and (c) DO‐LCO electrodes. Ex situ sXAS results for the O K‐edge obtained in TFY mode for the d) LCO and (e) DO‐LCO electrodes.

The operando differential electrochemical mass spectrometry (DEMS) measurement and soft X‐ray absorption spectroscopy (sXAS) were further employed to analyze the effect of Li^+^ diffusion optimization on the structure evolution. First, the DEMS measurement results indicate that no oxygen release was observed in DO‐LCO during the entire charging and discharging process, suggesting that the diffusion optimization of Li^+^ and the regulated structural stress stabilized the lattice oxygen skeleton.^[^ ^15^ ^]^ However, the uneven distribution of Li^+^ in LCO is accompanied by the surface phase transition from layered to rock salt phase, which makes cobalt ions migrate to adjacent tetrahedron or octahedron positions and O^2−^ breaks free from the binding of TM, leading to the release of a large amount of oxygen. Moreover, the formation of rock salt phase disrupts the surface structure of the material, leading to further erosion of the electrolyte to the bulk material, which further brings more serious side reactions and a large amount of CO_2_ release. The small amount of CO_2_ generated by DO‐LCO also indicates that the optimized structural stress regulates interface side reactions (**Figure** **4**a). Then, from the sXAS results, the pre‐edge peaks (whose shape changes with Co gaining and losing electrons) below 533 eV correspond to the unoccupied O 2*p* states originating from the covalent mixing of the O 2*p* and TM 3*d* orbitals in total electron yield (TEY) mode (Figure 4b,c).^[^ ^16^ ^]^ The pre‐edge features can be further distinguished by two peaks, characterizing the excitation from the O2*p* orbital to unoccupied t_2g_ (531 eV) and e_g_ orbitals (533 eV) of the TM.^[^ ^17^ ^]^ The difference in pre‐edge intensity between pristine and 3.0 V discharged states for DO‐LCO material is significantly lower than that of LCO, indicating that the homogenized Li^+^ concentration and reduced structural stress improve the reversibility of anionic redox. In addition, the peaks near 534 eV correspond to C–O groups and are usually related to the formation of cathode electrolyte interface (CEI).^[^ ^18^ ^]^ A peak corresponding to the interface side reaction appears when LCO material is discharged to 3.0 V (Figure 4b). On the contrary, no such peak was found in DO‐LCO, indicating that interface side reactions were suppressed (Figure 4c). However, it is not always reliable to determine the electronic structure of bulk materials using only the TEY model due to the interference of oxygen‐containing surface substances, such as inorganic (Li_2_CO_3_) and organics on the sample surface. Therefore, O sXAS in total fluorescence yield (TFY) mode are further studied. The peaks of O K‐edge XAS spectra could be fitted to Co^3+^(eg)‐O 2p hybridization peak located at 531.2 eV at the pristine state. For pristine state, no Co^4+^(eg)‐O 2p peak were observed in the sXAS spectra of both LCO and DO‐LCO electrodes (Figure 4d,e). Subsequently, Co^4+^(eg)‐O 2p peaks obviously emerged with the highly extraction of Li^+^ in LCO electrode at 4.6 V. This could be caused by the drastic Co‐O deterioration under high voltage, further accumulating the irreversible Co_3_O_4_ phase.^[^ ^17^, ^18^ ^]^ However, DO‐LCO cathode showed less accumulation of Co^4+^ at this TFY probing depth, effectively inhibiting the occurrence of phase transitions. In addition, X‐ray photoelectron spectroscopy (XPS) fitting analysis was performed on LCO and DO‐LCO samples at 4.6 V charging state to further obtain the contents of Co^3+^ and Co^4+^. In order to better show the surface of LCO and DO‐LCO materials, the surface of the two materials were etched at 20 and 200 nm, respectively, corresponding to the cathode electrolyte interface and LGTP on the surface. The relative intensity of the shake‐up satellite observed in Figure S25 (Supporting Information) is an essential parameter to check the evolution of the cobalt oxidation state. It is obvious that the strong decrease of the relative satellite area (from 31.41% to 29.33%) observed in Figure S25 (Supporting Information), together with the strong broadening of the main peak, can be attributed to an oxidation process of Co^3+^. It can be seen from Figure S25a (Supporting Information) that more Li^+^ is removed from the surface of LCO when charged to 4.6 V, accompanied by more Co^3+^ being oxidized to Co^4+^, which is consistent with the results of XAS. The high Co^4+^ content further leads to irreversible phase transition, which will cause serious damage to the structure of the material. Meanwhile, except for a slight difference in the pre‐edge peak intensity when DO‐LCO undergoes initial charging and discharging process, the overall spectrum is consistent with the state without charge, further showing a highly reversible structure (Figure 4e). The above results clearly show that the optimization of Li^+^ diffusion reduce the structural stress and inhibited the phase transition of the material, thus leading DO‐LCO shows an excellent long cycle stability at 4.6 V.

Electron paramagnetic resonance (EPR) spectra are further used to study the relationship between optimized Li^+^ diffusion and structural stability. A new sharp signal with an effect g‐value of 2.007 at 3380 G is observed for the charged LCO and DO‐LCO (4.6 V). Such signal can be assigned to oxidized oxygen species O_2_^n−^ (Figure S26, Supporting Information). Especially, only O_2_^3−^ and superoxide O_2_^−^ have a single unpaired electron (*S* = 1/2), which can be detected in EPR, while peroxo O_2_^2−^ has an even number of paired electrons (*S* = 0), resulting in EPR silence.^[^ ^19^ ^]^ The single electron content of O_2_^3−^ and superoxide O_2_^−^ in DO‐LCO is significantly higher than that of LCO charged to 4.6 V. However, it is worth noting that the lower superoxide O_2_^−^ in DO‐LCO indicates that its anion redox reaction is highly reversible after one cycle, and the presence of superoxide free radicals is likely to lead to the release of oxygen in LCO, which is consistent with the DEMS results (Figure 4a). Both DEMS and EPR results indicate that uniform Li^+^ concentration optimizes the structural stress and further stabilizes the oxygen skeleton during the charging and discharging process.

To further verify the effect of Li^+^ diffusion optimization and structure stress on the side reactions occurring at the interface between the cathode and electrolyte, the pristine and charged states of the two cathodes were tested using ex situ XPS measurement. The detailed composition of LCO and DO‐LCO surface was obtained (Figure S27 and S28, Supporting Information). The O 1s spectra are dominated by LCO lattice oxygen (530.28 eV), C–O (532.67 eV), and CO_3_ (530.90 eV) peaks before cycling, whereas the functional groups of CEI components, such as ROLi (531.41 eV) and Li_x_PF_y_O_2_ (534.48 eV), are detected after initial charge cycling.^[^ ^20^ ^]^ Considering the limited probing depth of XPS (<10 nm), the thickness of CEI could be calculated by the relative intensity change of the lattice oxygen peak originating from the cathode (the detailed calculation process is shown in the Supporting Information, and the calculation results are shown in Table S9, Figures S27,S28, Supporting Information).^[^ ^21^ ^]^ The CEI thickness of LCO and DO‐LCO electrodes is 24 Å and 9.7 Å at the charged state, respectively. In addition, the peak area of C–O and C═O account for 19.1% and 17.7% in DO‐LCO which are less than that of LCO, further suggesting that the optimized structural stress can effectively inhibit the occurrence of interface side reactions.

Figures S29,S30 (Supporting Information) show the in situ Raman results of LCO and DO‐LCO. The two characteristic peaks at 485 and 595 cm^−1^ are related to the changes of O–Co–O bending (Eg) and Co–O stretching (A1g) of LCO and DO‐LCO, and these two characteristic peaks are closely related to the structural changes during the charge and discharge process.^[^ ^22^ ^]^ Figure S29 (Supporting Information) clearly suggest that the Eg peak of LCO electrode disappears at charge and discharge transition state, while the Eg peak of DO‐LCO electrode still exists in the whole charge and discharge process. This can be attributed to the homogenized Li^+^ concentration which reduce the structure stress, preventing the irreversible fracture of Co–O and O–Co–O bonds, and further hindering the collapse of deep structure under highly oxidized state. Meanwhile, LCO material has an obvious peak at 900 cm^−1^ corresponding to the decomposition of the electrolyte, while the peak does not appear in DO‐LCO material. This is because that the reduced structural stress suppresses the electrolyte decomposition and hinder the occurrence of interface side reactions (Figures S27,S28, Supporting Information).^[^ ^23^ ^]^

The microstructural change of pristine LCO and DO‐LCO electrodes after 100 cycles under the voltage of 3.0–4.6 V are observed by the high resolution transmission electron microscope (HRTEM) to further explore the effect of diffusion optimization on the structural stability (Figures S32,S33, Supporting Information). Clear and orderly lattice stripes was observed in DO‐LCO particles, which contrasted with the severe degradation of the surface structure of LCO particles (lattice stripe distortion). To clearer explore the impact of Li^+^ diffusion optimization on the structural stability, the microstructure changes of LCO and DO‐LCO electrodes were observed by SEM measurements after different cycles at a voltage of 3.0–4.6 V (Figures S34,S35, Supporting Information). It can be clearly observed that a few cracks appear in LCO after 10 cycles, the cracks almost become more severe throughout the entire particle after 40 and 80 cycles. Notably, the particles began to slip after 100 cycles, and the slip of the particles became more obvious at 150 cycles, which may be due to the phase transition and near surface structure stress caused by the uneven distribution of Li^+^ between the bulk and the surface at highly delithiated state. On the contrary, the surface and interior of the cyclic DO‐LCO electrode maintained mechanical integrity of morphology, without obvious microcracks after 10 cycles, 40 cycles, 80 cycles and even 100 cycles and 150 cycles, further shown that the optimization of Li^+^ diffusion makes the structural stress distribution more uniform and successfully stabilizes the material structure. Notably, to make the results more convincing, we selected four different regions for cross‐sectional scanning of each sample. The cross‐sectional SEM images of LCO and DO‐LCO materials after 100 cycles can also support the conclusion that the diffusion optimization of Li^+^ can stabilize the material structure and suppress phase transition (Figure S36,S37, Supporting Information).

**Figure 5:**
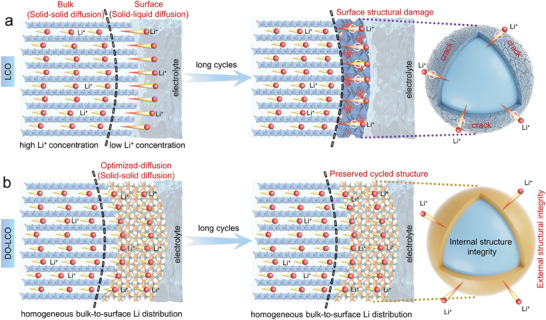
Schematic illustration of Li diffusion environments and long‐cycled structure evolutions for the ( a) LCO and (b) DO‐LCO samples.

To better clarify the relationship between Li^+^ diffusion, structural stress and electrochemical properties, a schematic diagram is applied to summarize the evolution mechanism of the structure (**Figure** **5**). It can be seen from Figure 5a that the de‐intercalation of Li ions is accompanied by solid–solid diffusion in the bulk structure and solid‐liquid diffusion when in contact with the electrolyte. The Li^+^ on LCO surface exhibits rapid diffusion kinetics at the solid–liquid interface, while the transport at the solid–solid interface exhibits slower kinetic behavior. Accompanied by repeated de‐intercalation of Li ions, such different diffusion can result in an inhomogeneous bulk‐to surface Li distribution, leading to a large structure stress at the near surface (where shows the largest Li concentration difference). The diffusion‐induced structure stress can cause strains accumulation and microcracks near the surface. It further leads to the collapse of the overall structure, directly destabilize the Li‐storage structure of LCO upon long cycles.

In contrast, via constructing an artificial diffusion‐optimized solid–solid Li diffusion environment on LCO's surface that is the same as the Li^+^ diffusion environment inside the bulk, which is equivalent to the first diffusion of Li^+^ to LGTP and then to the liquid electrolyte when Li^+^ are removed from the surface of LCO, a homogeneous bulk‐to‐surface Li distribution can be achieved to reduce the structural stress caused by different diffusion environments of Li^+^ upon long cycles (Figure 5b). The reduced structural stress preserves the cyclic structure from multiple perspectives, such as stabilizing the lattice oxygen structure framework, reducing oxygen release, suppressing interface side reactions and phase transitions, ultimately achieving superior long‐cycled stability.

## 3 Conclusion

In summary, the construction of an artificial solid–solid Li diffusion environment on LCO's surface successfully optimizes the Li^+^ diffusion behavior. Optimized‐diffusion drives a homogeneous bulk‐to‐surface Li distribution, reduces the structure stress of the material, ultimately achieves structural stability and excellent long cycle electrochemical performance. A long lifespan DO‐LCO (exceed 600 cycles) with a good capacity retention (>80%) and 0.11 mV per cycle voltage decay were achieved. Through systematic characterizations, we determine that these superb electrochemical properties are due to the reduced structural stress enabled by homogenized Li^+^ concentration, which in turn prevent oxygen release, stable the oxygen lattice framework, inhibits the occurrence of side reactions at the interface and finally achieving an extremely long cycle life. More importantly, the proposed diffusion‐optimized strategy supplies a general guidance for designing high‐energy‐density cathode materials.

## Conflict of Interest

The authors declare no conflict of interest.

## Supporting information

Supporting Information

## Data Availability

The data that support the findings of this study are available from the corresponding author upon reasonable request.
