# Supplementary material for: Diffusion‐Optimized Long Lifespan 4.6 V LiCoO2: Homogenizing Cycled Bulk‐To‐Surface Li Concentration with Reduced Structure Stress
Source: Adv Sci (Weinh). 2024 Jan 30;11(14):2308258. doi: 10.1002/advs.202308258 (PMC11005714; doi:10.1002/advs.202308258)
Supplement: Supplementary file 1 — Supporting Information [file ADVS-11-2308258-s001.pdf]

ADVANCED SCIENCE
Open Access

Supporting Information

for *Adv. Sci.*, DOI 10.1002/advs.202308258

Diffusion-Optimized Long Lifespan 4.6 V $LiCoO_2$: Homogenizing Cycled Bulk-To-Surface Li Concentration with Reduced Structure Stress

*Kang Wu, Peilin Ran, Baotian Wang, Fangwei Wang, Jinkui Zhao and Enyue Zhao**

Diffusion-Optimized Long Lifespan 4.6 V $LiCoO_2$: Homogenizing Cycled Bulk-to-Surface Li Concentration with Reduced Structure Stress

Kang Wu, Peilin Ran, Baotian Wang, Fangwei Wang, Jinkui Zhao, Enyue Zhao*

K. Wu, P. Ran, F. Wang, J. Zhao, E. Zhao*

Songshan Lake Materials Laboratory, Dongguan 523808, China

E-mails: eyzhao@sslab.org.cn

K. Wu, P. Ran, F. Wang, J. Zhao

Beijing National Laboratory for Condensed Matter Physics, Institute of Physics, Chinese Academy of Sciences, Beijing 100190, China

B. Wang

Institute of High Energy Physics, Chinese Academy of Sciences, Beijing 100049, P. R. China.

B. Wang, F. Wang

Spallation Neutron Source Science Center, Dongguan 523803, Guangdong, China.

Methods

Preparation DO-LCO samples

To achieve uniform Li^+ transport between the bulk and surface, we have constructed an artificial solid-solid Li diffusion environment (Li^+ diffusion coefficient of $Li_{1.5}Ga_{0.5}Ti_{1.5}(PO_4)_3$ (LGTP) is 7.4*10^-13 cm^2/s) on the surface of LCO that is similar to the Li^+ transport environment inside the bulk (Li^+ diffusion coefficient of LCO is 2.7*10^-12 cm^2/s). In this work, we select the typical Li-ion conductor LGTP as the research model, and highlight the role of cycled Li distribution and diffusion-induced stress on the Li-storage structure stability.

The $LiCoO_2$ used in this work was purchased from Hefei Kejing Material Technology Co., Ltd. with a purity of 99.5%. Different proportions of solid electrolyte $Li_{1.5}Ga_{0.5}Ti_{1.5}(PO_4)_3$ (LGTP) are introduced to the surface of LCO material to change the environment of Li^+ transport. Firstly, $LiNO_3$, $Ga(NO_3)_3$, $C_{12}H_{28}O_4Ti$, $NH_4H_2PO_4$ with the molar ratio Li:Ga:Ti:P of 1.5:0.5:1.5:3 were first dissolved in ethanol, citric acid was then added to the above solution and mixed thoroughly for 5 h at 50 °C to obtain LGTP sol solutions. Next, LCO powder was mixed with LGTP sol solutions with the amount of LGTP being 0.3 wt%, 0.5 wt% and 0.7 wt% of LCO powder, respectively. The mixtures were stirred constantly at 80 °C until the solvents have evaporated. The thus obtained precursor gels were dried at 130 °C for 12 h in air, and then calcined at 900 °C for 10 h to achieve the surface of solid electrolyte.

Preparation LGTP samples

In order to further compare the Li+ diffusion ability of LCO and surface LGTP, we synthesized pure LGTP material through sol-gel process, and the specific synthesis method is as follows: stoichiometric amounts of $LiNO_3$, $Ga(NO_3)_3$, $C_{12}H_{28}O_4Ti$ and $NH_4H_2PO_4$ were used. $LiNO_3$, $Ga(NO_3)_3$, $C_{12}H_{28}O_4Ti$, $NH_4H_2PO_4$ with the molar ratio Li:Ga:Ti:P of 1.5:0.5:1.5:3 were first dissolved in ethanol, citric acid was then added to the above solution and mixed thoroughly for 5 h at 50 °C to obtain LGTP sol solutions. The gel was dried at a temperature of 80°C and calcined at 700°C in air to obtain LGTP material.

Materials characterizations

Laboratory characterizations

The crystal structures of synthetic samples were analyzed using X-ray powder diffraction (XRD) on a Persee XD2 diffractometer with Cu Kα radiation and angle coverage of 10°-80°.

The element contents and dissolved Co contents in electrolyte after cycles were detected using an inductively coupled plasma (ICP) spectrometer. Sample surface element valence states and compositions were measured by X-ray photoelectron spectroscopy (XPS) using a Thermo Fisher ESCALAB XI+. Scanning electron microscopy (SEM) characterization of the samples were carried out by using a Gemini 300 equipment. Sample elemental mapping images and microstructural change were obtained by high resolution transmission electron microscopy (HRTEM) on a JEM-F200. The relationship between Li^+ diffusion and structural stability was evaluated by ex-situ electron spin resonance (EPR) spectroscopy (EMXplus). For *ex-suit* testing, cathode materials were charged to 4.6 V, washed with dimethyl carbonate (DMC) and then scraped off from the current collectors. STEM (Spectra 300) equipped with a field emission gun at 300 kV was utilized to confirm the atomic arrangement and structure of the samples. GPA method is proposed to extract the 2D relative strain maps from HAADF-STEM image and analyze the internal and external stress of highly delithiated LCO.

***In-situ* XRD and *in-situ* Raman**

In-situ XRD was performed on a Bruker D8-ADVANCE X-ray diffractometer using a Cu target under 45 kV and 40 mA. The data were collected at 2θ 3° per minute and the 2θ angle range is 10°–50°. In order to allow X-ray to penetrate the *in-situ* cell, Be metal was designed as a diffraction window. For the *in-situ* Raman (on Horiba LabRam HR Evolution) measurements, a thin quartz window (0.5 mm thick) is fixed on the top of the battery and a small hole is punched in the center of the separator/lithium foil to collect laser and Raman signals. The charge/discharge current density is 27.4 mA g^{-1} in a voltage range of 3.0–4.6 V for the *in-situ* XRD and *in-situ* Raman tests.

***In-situ* differential electrochemical mass spectrometry (DEMS)**

Differential electrochemical mass spectrometry experiments were carried out on a PM-DEMS instrument in a customized battery case (ECC-DEMS). A slurry consisting of 80 wt.% active materials, 10 wt.% polyvinylidene fluoride (PVDF) binder, and 10 wt.% conductive carbon was coated on a 12-mm-diameter aluminum foil collector. Lithium metal was used for the anode electrode. Glass fiber was used as the separator. The cell was charged/discharged at 3.0–4.6 V with a current density of 27.4 mA g^{-1}. High-purity argon (99.999 %) was used as the carrier at a flow rate of 0.7 sccm.

***Ex-situ* soft X-ray absorption spectroscopy (sXAS)**

The sXAS data were collected on the beamline BL20A1 in National Synchrotron Radiation Research Center (NSRRC). The beamline covers the spectral range from 60 eV to 1250 eV, with an average energy resolving power of 5000. sXAS can be detected to depths of several nanometers in total electron yield (TEY) mode and hundreds of nanometers in total fluorescence yield (TFY) mode. Thus, the TEY model is surface sensitive, while the TFY model is used to evaluate the electronic structure of the bulk material.

Electrochemical measurements

CR2032 coin cells with liquid electrolyte were assembled using an electrolyte composed of 1.2 M $LiFP_6$ in ethylene carbonate (EC) and diethyl carbonate (DEC), glass fiber separators, and Li as the counter electrode, in high-purity argon atmosphere. LCO and DO-LCO active materials, super P conductive additive, and poly (vinylidene fluoride) were mixed in 80:10:10 ratio to obtain a uniform slurry with a thickness of 150 μm, which was then dried at 120 °C for 12 h in a vacuum oven, and the mass loading of both active substance is 1.4 mg/cm^2 approximately. Galvanostatic charge-discharge and Galvanostatic Intermittent Titration Technique (GITT) tests were conducted on an automatic galvanostat (NEWARE) in the voltage window of 3.0-4.6 V at various current densities (1 C=274 mA g^{-1}). The cyclic voltammetry (CV) measurement was employed through an electrochemical workstation (PGSTAT302N, Autolab).

COMSOL simulation

The finite element analysis is carried out by the commercial software COMSOL Multiphysics. A three-dimensional electric field and ion distribution model based on COMSOL was established to elucidate the distribution of ions in different structures (LCO and DO-LCO), coupled with solid mechanics to calculate stresses due to uneven delithium and insertion to predict particle damage. The model uses a 4 μm cube to simulate nanoparticles and a comparison sample of modified particles covered with LGTP (0.1 μm). The model is considered as a multi-physics coupling of the Nernst-Planck equation for the mass transport of all particles. The exchange current density at the electrode surface satisfies the Butler-Volmer equation with an exchange current density of 100 A m^{-2}, a transfer coefficient of 0.5 and the electrolyte current density is 1 mA cm^{-2}.

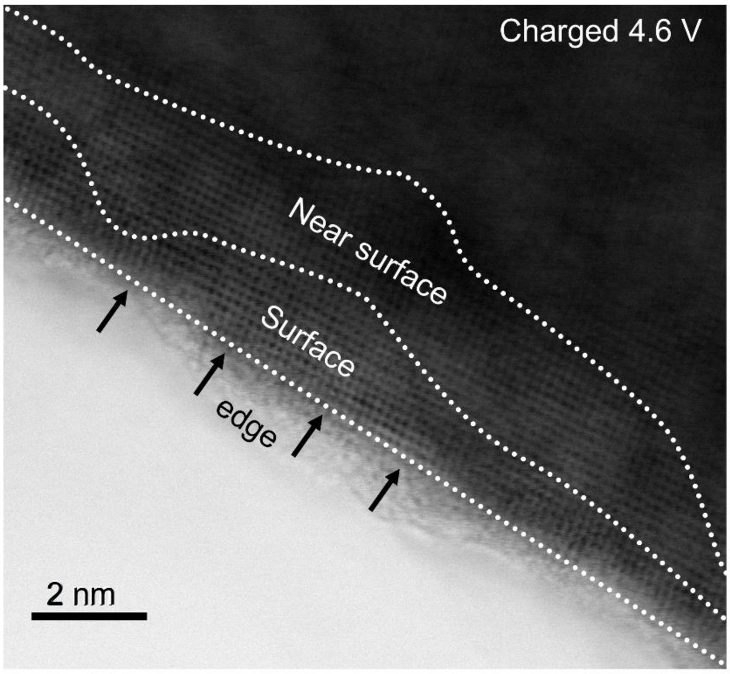

Figure S1. The STEM image for the 4.6 V-charged LCO.

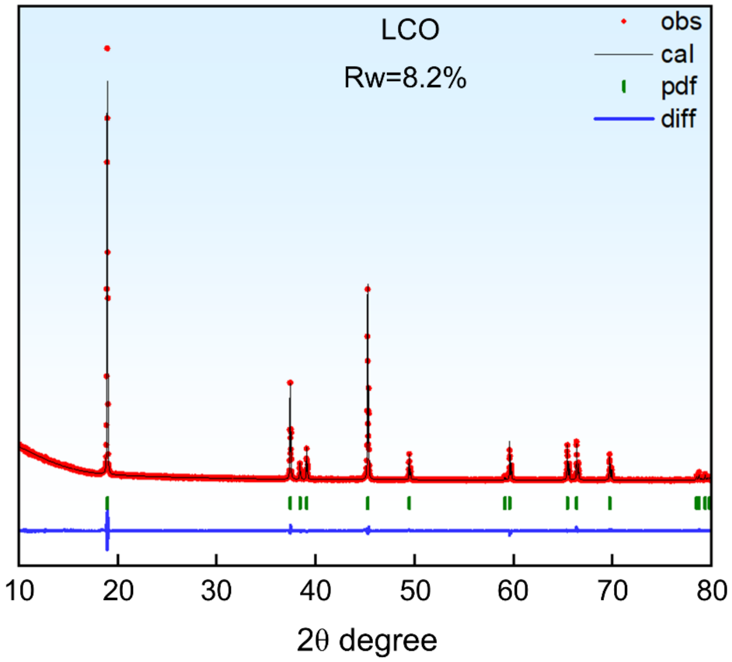

Figure S2. XRD data and corresponding refinement results for LCO sample.

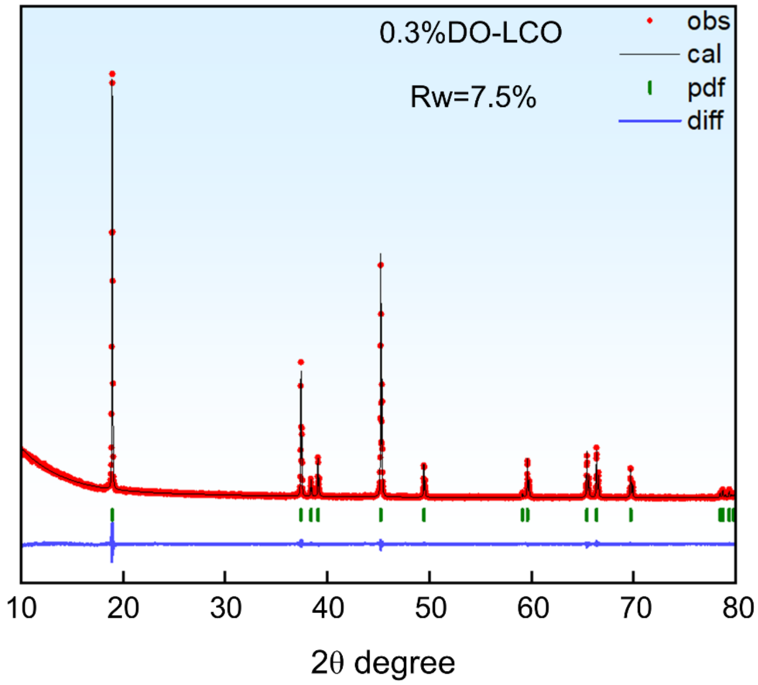

Figure S3. XRD data and corresponding refinement results for 0.3% DO-LCO sample.

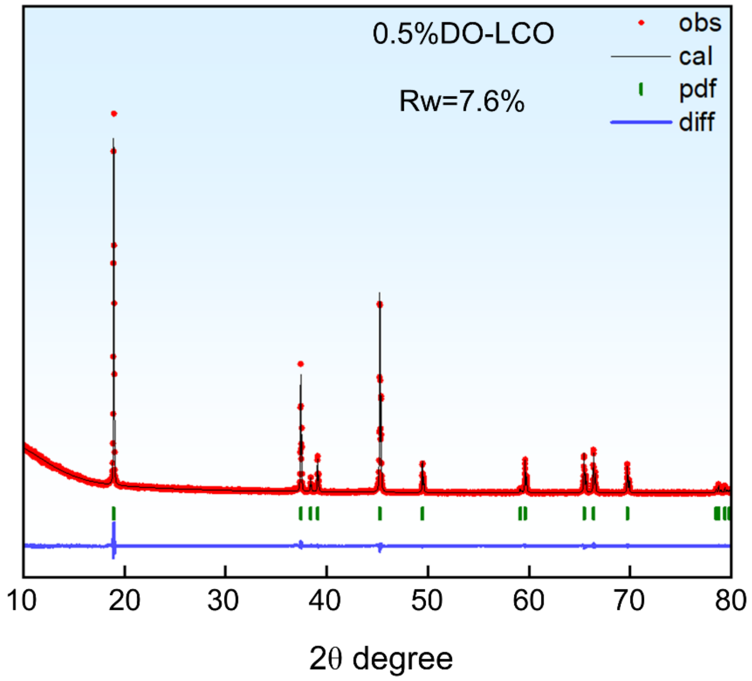

Figure S4. XRD data and corresponding refinement results for 0.5% DO-LCO sample.

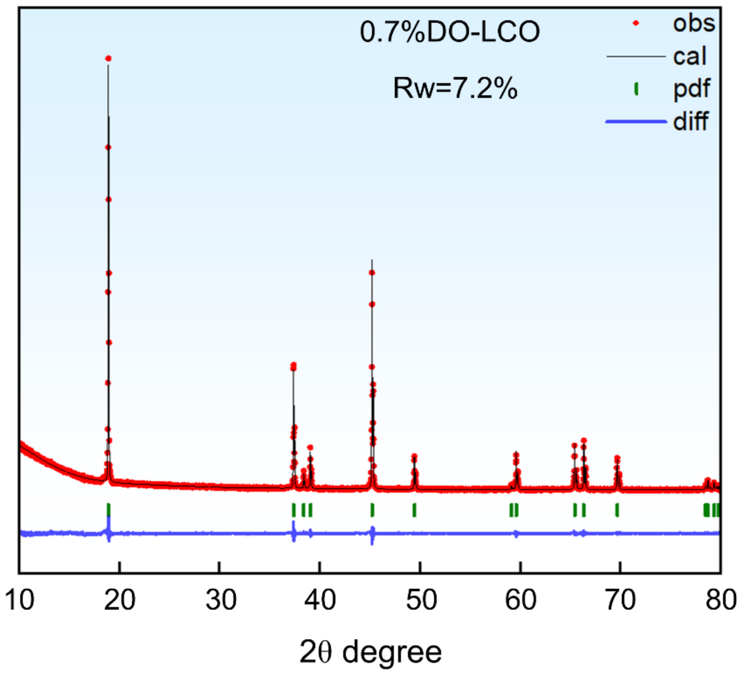

Figure S5. XRD data and corresponding refinement results for 0.7% DO-LCO sample.

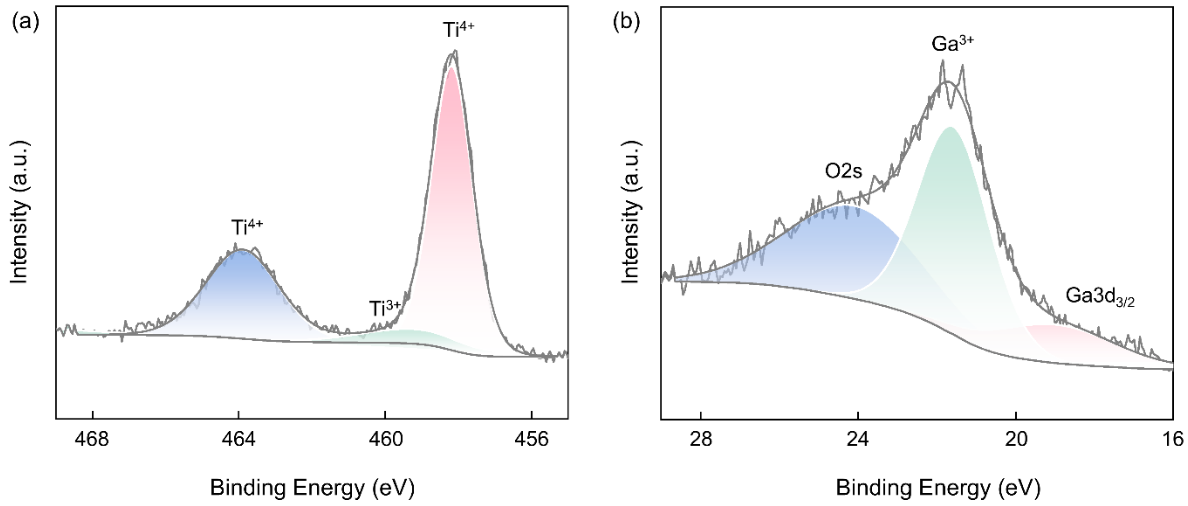

Figure S6. The Ga^{3+} and Ti^{4+} XPS spectrogram for DO-LCO material.

XRD was firstly used to evaluate the layered crystal structure of LCO and diffusion optimized electrodes (Figure S2-S5). All samples can be described using a well-developed layered structure with an emblematical group space of R-3m (No. 166). In addition, the Rietveld refinement calculated crystallographic parameters are summarized in Table S2-S5. It can be seen from the refinement results that the lattice parameter *c* gradually increases with the increase of solid electrolyte content, mainly because Ga^{3+}/Ti^{4+} with small radius are doped in the transition metal (TM) layer shortening the TM layer distance during the high temperature sintering process. In addition, we also performed XPS analysis for two elements to further

verify that Ti^{4+} and Ga^{3+} were successfully doped into the material (Figure S6).[S1]

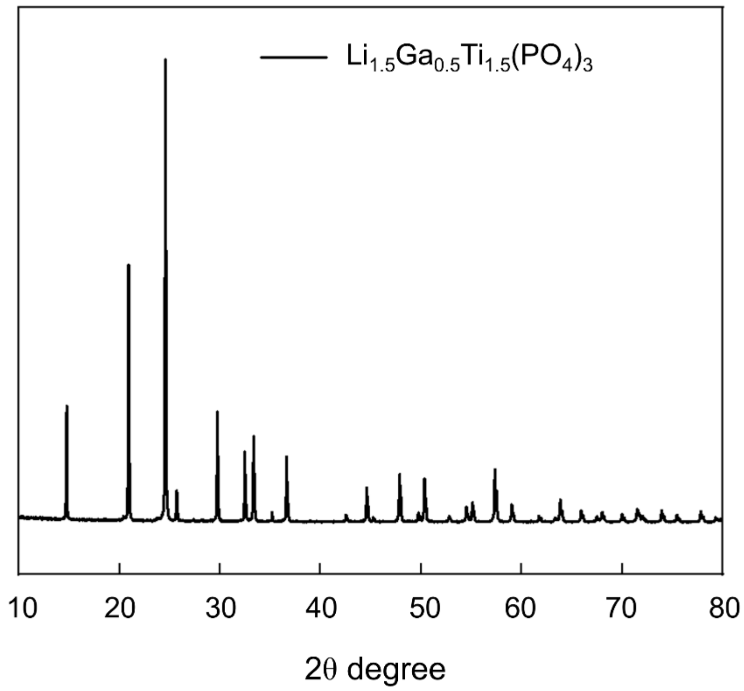

Figure S7. The XRD pattern for the LGTP material. XRD results show that LGTP has been successfully synthesized.

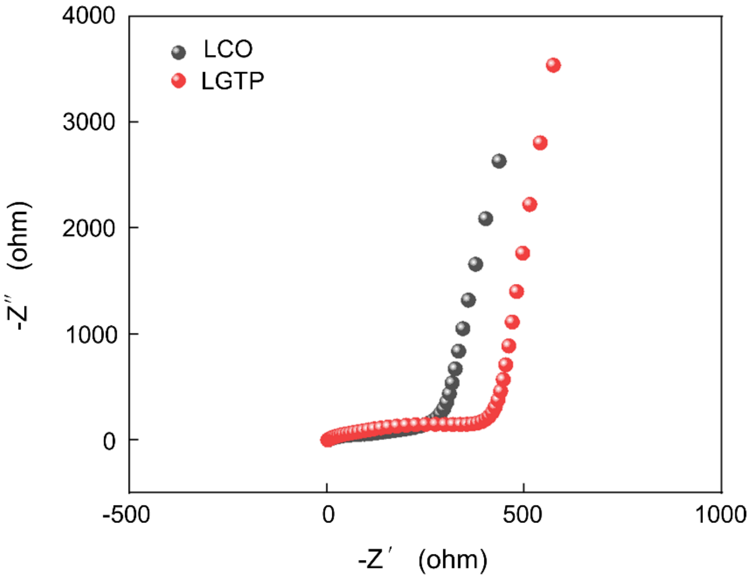

Figure S8. Nyquist plots of LCO and LGTP materials.

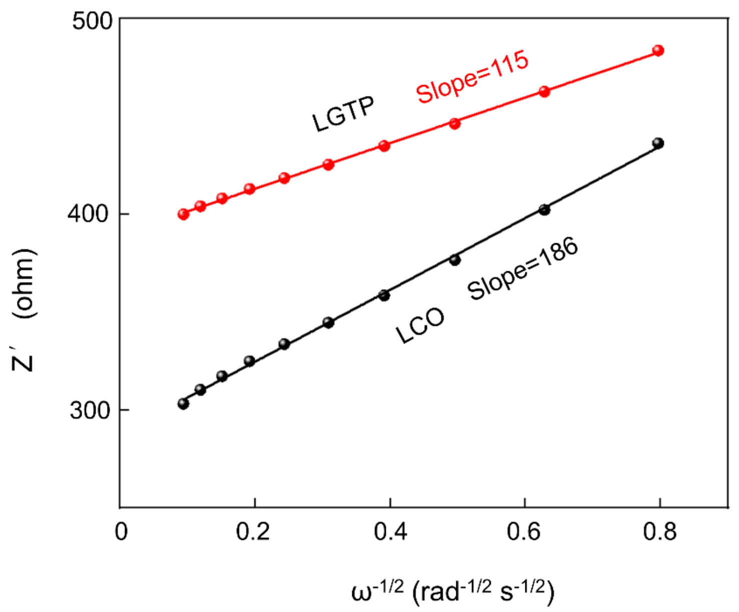

Figure S9. The relationships between Z' and $\omega^{-1/2}$ of LCO and LGTP.

The resistance and the Li^+ diffusion coefficient of LCO and LGTP materials was obtained through electrochemical impedance spectroscopy (EIS). The specific impedance spectroscopy (Figure S8) and the calculation results of the Li^+ diffusion coefficient are as follows: According to Equation (1):

$$D_{Li+} = R^2T^2/2A^2n^4F^4C^2\sigma^2 \qquad (1)$$

where T, F, and R, are absolute temperature, Faraday's constant, and gas constant, respectively. A and C are the area of electrode and Li ion molar concentration, respectively. Warburg factor (σ) can be determined by Equation (2):

$$Zreal = Re + Rct + \sigma\omega^{-1/2} \qquad (2)$$

According to formula 2, the calculated σ value of LCO and LGTP are 186 and 115, so the Li^+ diffusion coefficient (D_{Li+}) corresponding to LCO and LGTP are 2.7*10^-12 cm^2 s^{-1} and 7.4*10^-13 cm^2 s^{-1}, respectively. The Li ion diffusion coefficients in LCO and LGTP calculated by EIS are consistent with those reported in previous literatures.[S2] The diffusion coefficient of Li^+ in LGTP is smaller than that of LCO, which indicates that the LGTP layer with slow solid-solid diffusion environment close to the bulk can achieve a homogeneous bulk-to-surface Li distribution upon cycling.

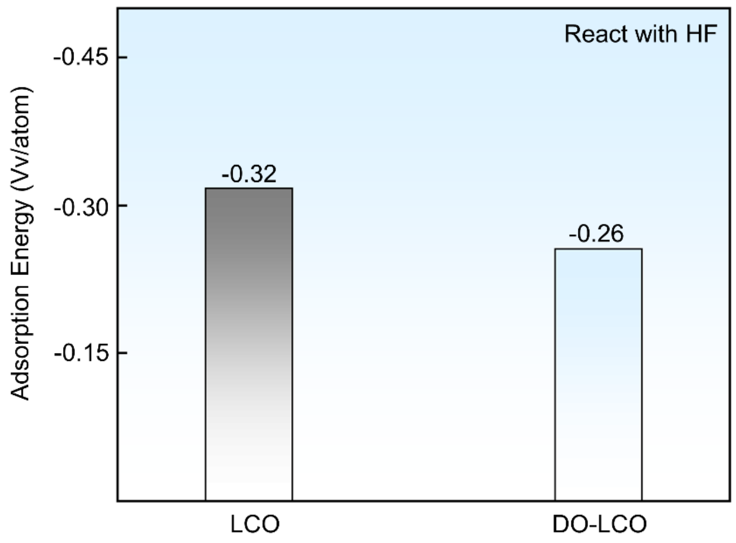

Figure S10. Formation energy of the reaction for LCO and LGTP with HF.

In order to better clarify the relationship between solid electrolyte and the excellent electrochemical properties associated with structural integrity, the reaction energies between LCO/solid electrolyte and HF in the electrolyte are calculated. The results show that the binding energy between solid electrolyte and HF is smaller than that of LCO, indicating that LGTP can prevent the corrosion of the electrolyte, thus inhibiting the irreversible phase transition and structural collapse during the long cycles.

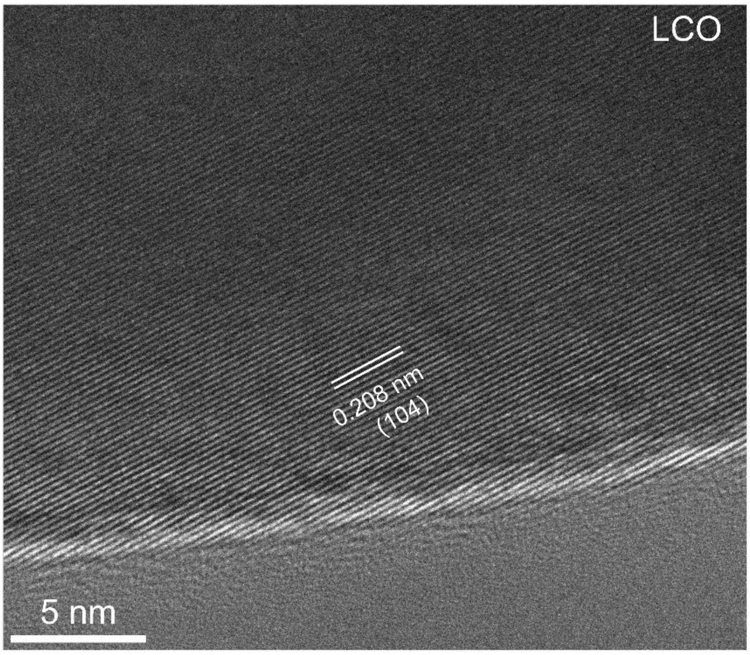

Figure S11. The TEM image of LCO.

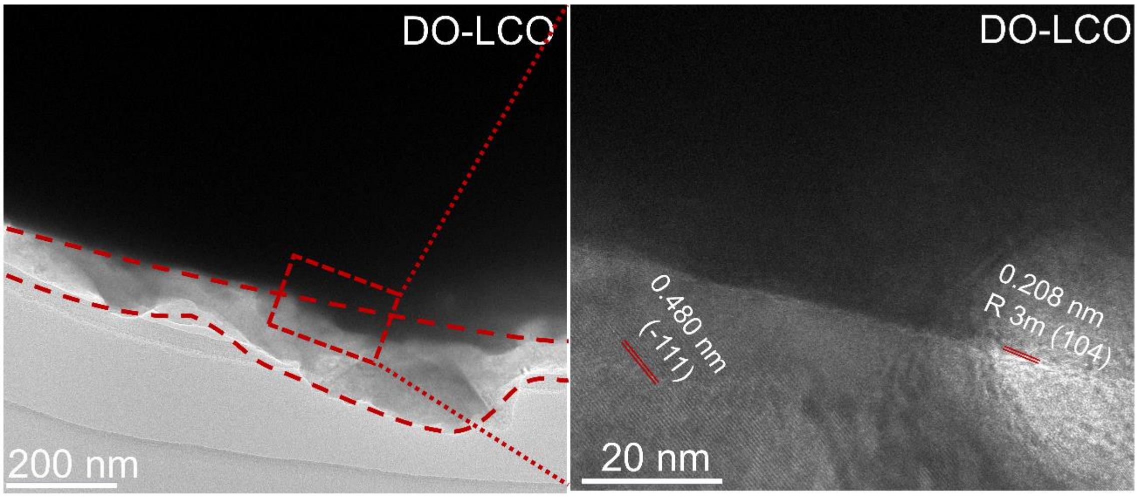

Figure S12. The TEM image of DO-LCO.

It can be seen from Figure S11 that LCO exhibits relatively regular lattice stripes accompanied by (104) crystal planes with a crystal plane spacing of 0.208 nm. Notably, it clearly confirms that the LGTP coating layer (about 200 nm) is well coated onto LCO in Figure S12. Besides, two kinds of different lattice fringes, the interplanar spacing values of 0.480 and 0.208 nm belong to the (-111) plane of LGTP (pristine rhombohedral phase LGTP coating layer transformed into a spinel-like phase after the high-temperature sintering)[S3] and the (104) plane of LCO, respectively, were distinguished.

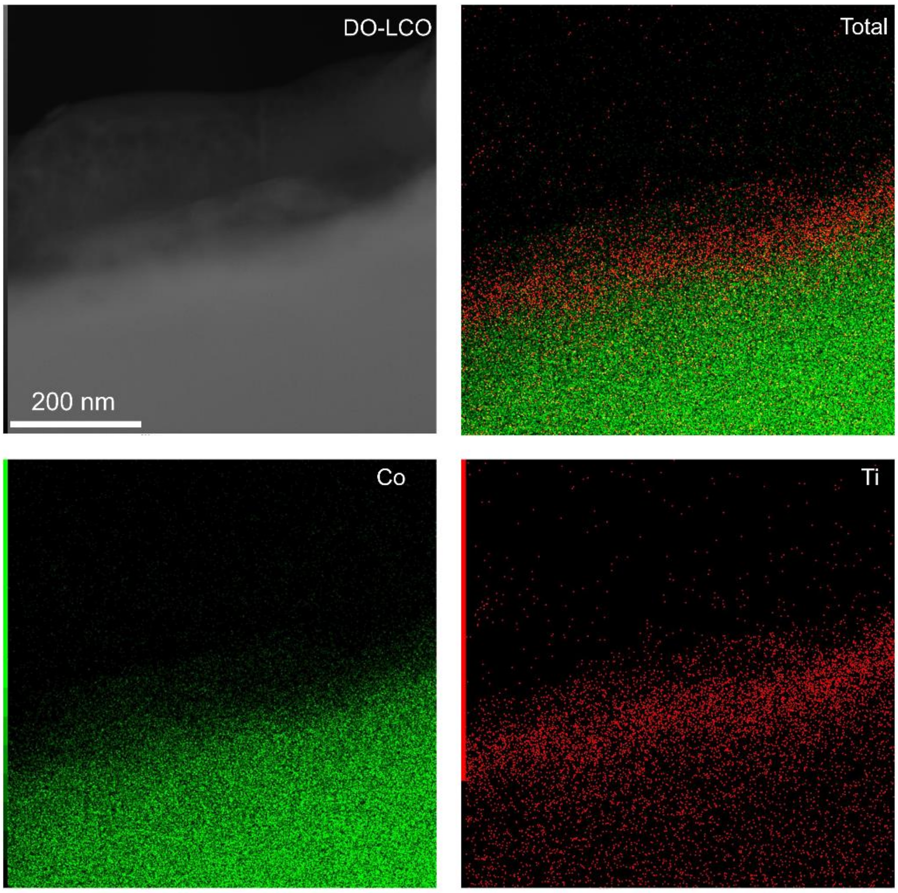

Figure S13. EDS overlay plot and single element distribution of Co and Ti elements in DO-LCO sample.

Energy dispersive spectroscopy (EDS) mapping was performed to obtain elemental distribution. Co is relatively evenly distributed in the bulk, while Ti elements are enriched on the surface of DO-LCO accompanied by a dense layer of about 100 nm. The EDS results well demonstrate our expected tailored solid electrolyte surface lattice structure.

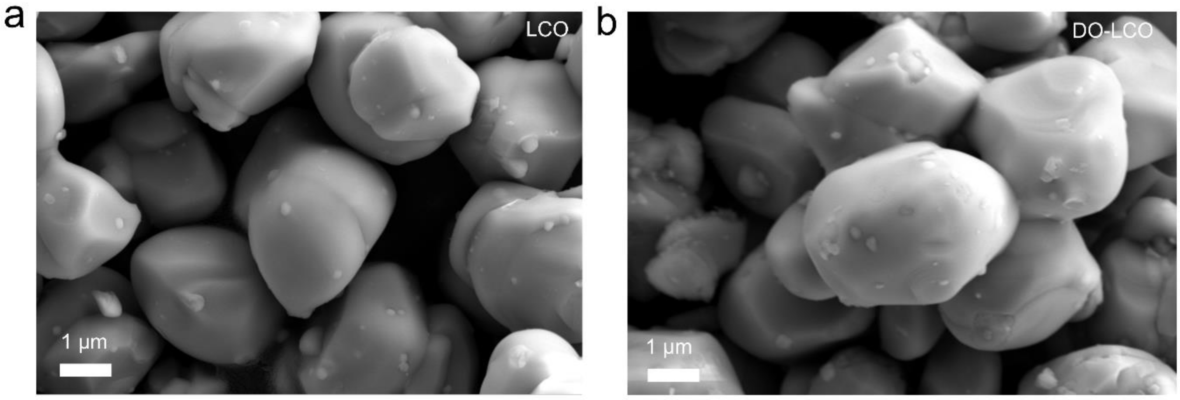

Figure S14. The scanning electron microscope (SEM) images of LCO and DO-LCO.

The morphologies of LCO and DO-LCO, both of which are all single crystal particles

with no difference in shape.

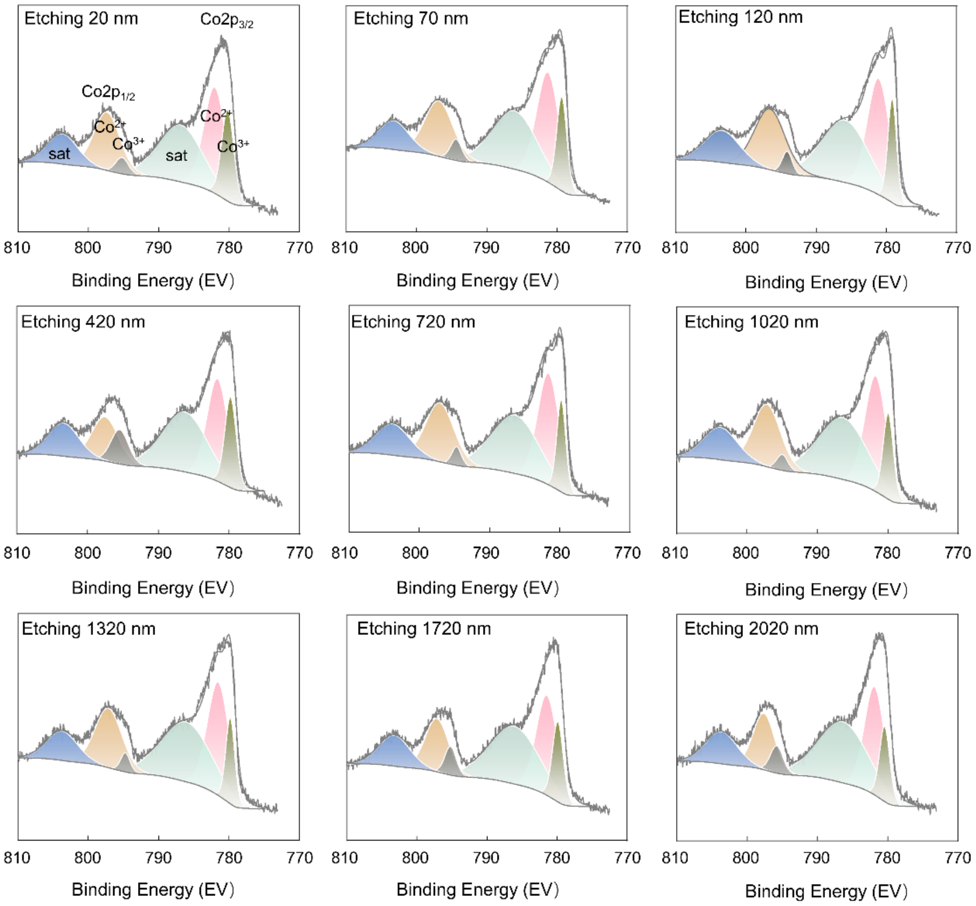

Figure S15. The Co 2p XPS for LCO at different etching depth.

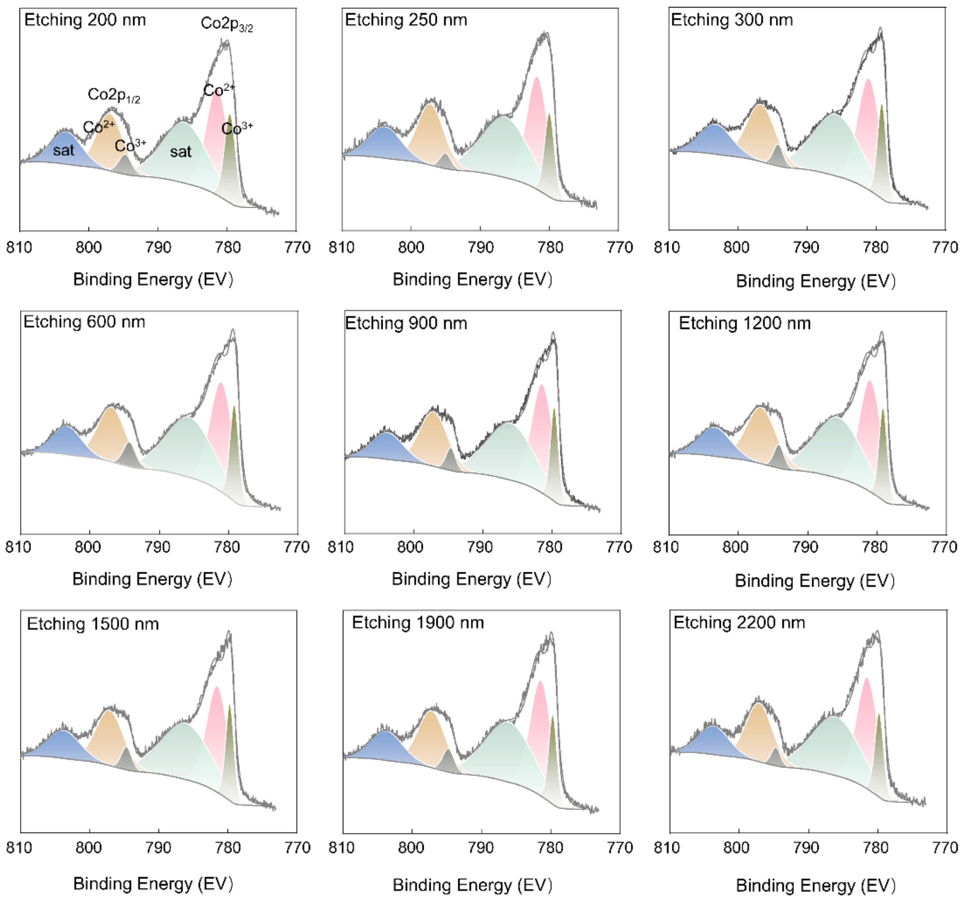

Figure S16. The Co 2p XPS peak for DO-LCO at different etching depth.

We conducted XPS etching of Li ions and Co ions on the 4.6 V charged state LCO and DO-LCO to further explore the in-depth chemical state of the electrodes. Due to the low concentration of Li^+ in 4.6 V highly delithiated state, XPS cannot detect the peak of Li 1s. However, the removal of Li^+ in the material is accompanied by the oxidation of Co^{3+}, so the distribution of Li^+ on the bulk and surface can be verified by the content of Co^{3+} and Co^{4+}. The relative intensity of the shake-up satellite observed in Figure S15 is an essential parameter to check the evolution of the cobalt oxidation state.[S4] In order to better show the surface of LCO and DO-LCO materials, the surface of the two materials were etched at 20 nm and 200 nm, respectively, corresponding to the cathode electrolyte interface and LGTP on the surface. It is obvious that the strong decrease of the relative satellite area (from 31.41% to 29.33%) observed in Figure S15, S16 and Table S6, together with the strong broadening of the main peak, can be attributed to an oxidation process of Co^{3+} at surface of LCO and DO-LCO. The above results indicate that the surface of LCO has a higher content of Co^{4+} than that of DO-LCO. It is worth noting that as the etching depth increases (surface to bulk), the area of satellite peaks in LCO

gradually increases, indicating that the surface and bulk exhibit a significantly inconsistent oxidation state of Co. However, the area of satellite peaks on the surface and bulk of DO-LCO changes relatively small, further confirming the uniform distribution of Li^+.

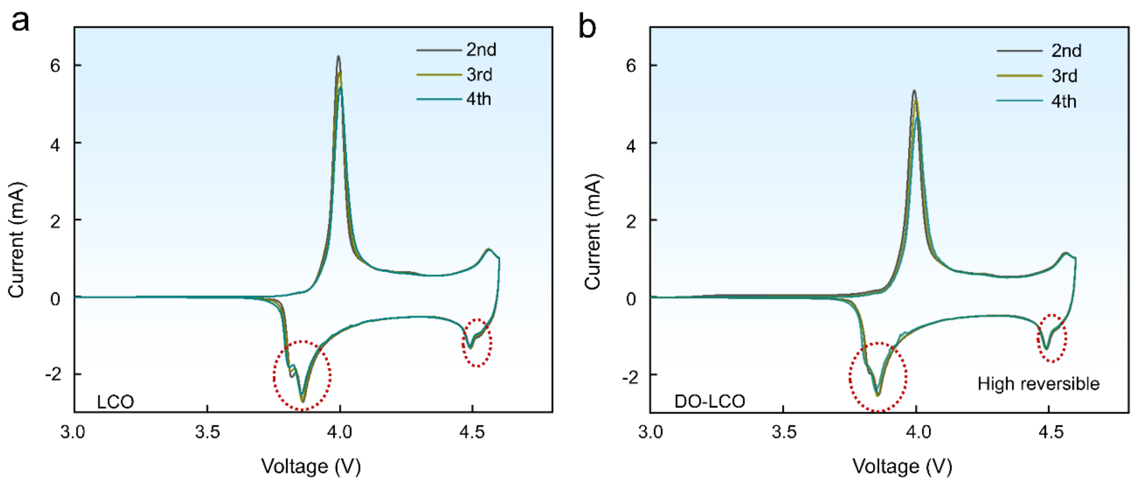

Figure S17. The CV curve of 2-4 cycles for LCO an DO-LCO materials.

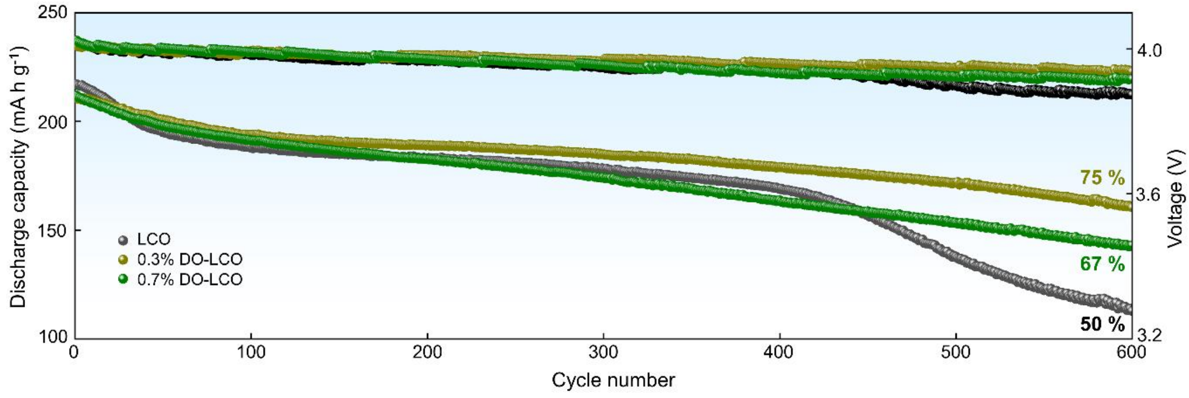

Figure S18. The long cycle and average voltage performance at 1 C of LCO, 0.3% DO-LCO and 0.7% DO-LCO materials.

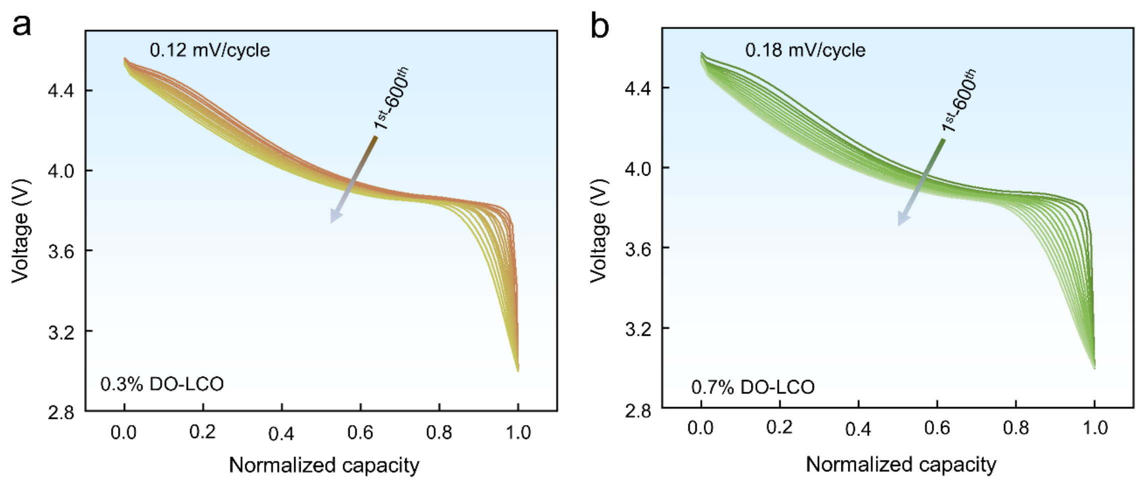

Figure S19. The variation of discharge curves upon cycling at 1 C for 0.3% DO-LCO (a) and

0.7% DO-LCO (b) cathodes.

In order to investigate the effect of Li^+ diffusion optimization on the electrochemical behavior of materials, all prepared materials were subjected to electrochemical performance testing. The initial discharge capacity is 212, 212, 212 mA h g^{-1} accompanied by capacity retention rates of 92%, 93% and 90% at 1 C for 0.3% DO-LCO, 0.5% DO-LCO and 0.7% DO-LCO samples, respectively. Notably, the capacity retention rate of 0.3% DO-LCO, 0.5% DO-LCO and 0.7% DO-LCO material is still as high as 75%, 80% and 67%, accompanied by the attenuation of LCO to only 50% at 1 C after 600 cycles, which suggests that diffusion optimization homogenizes Li^+ distribution, significantly improving the long cycle life of the material. Moreover, the average voltage attenuation rate at 1 C of 0.3% DO-LCO, 0.5% DO-LCO and 0.7% DO-LCO samples is only 0.018% (0.12 mV per cycle), 0.017% (0.11 mV per cycle) and 0.026% (0.18 mV per cycle) much lower than LCO of 0.035% (0.23 mV per cycle), further demonstrating that optimized Li^+ diffusion can suppress voltage attenuation. By evaluating various aspects of electrochemical performance of the prepared materials, we selected 0.5% DO-LCO material with high capacity, excellent cyclic stability and low voltage decay rate as representative material for subsequent electrochemical testing and structure characterization.

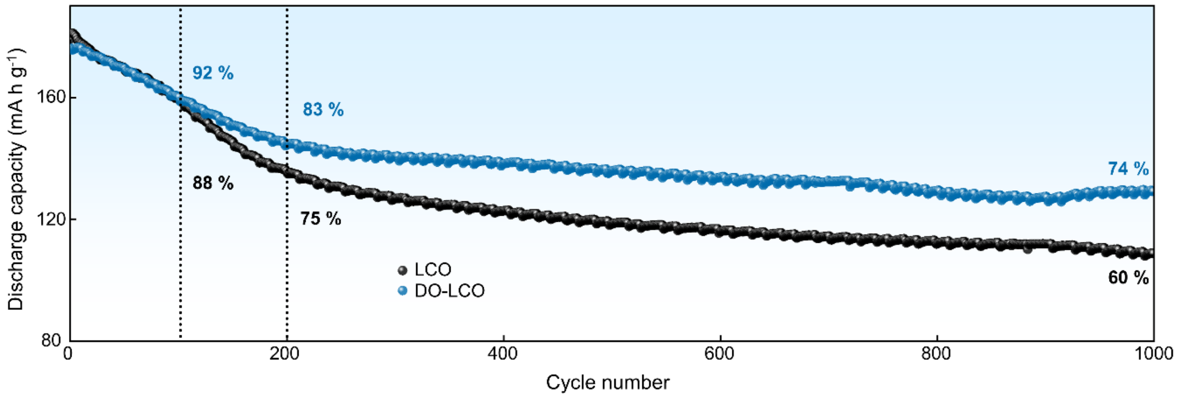

Figure S20. The long cycle performance at 10 C of LCO and 0.5% DO-LCO materials.

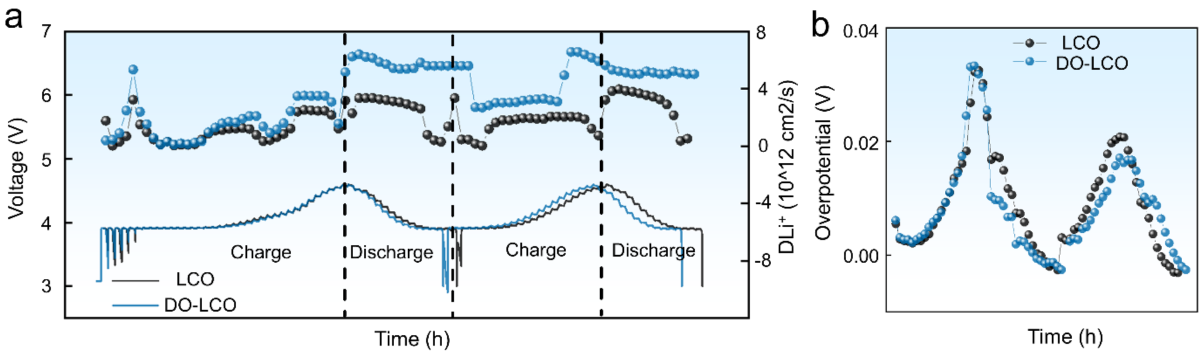

Figure S21. (a) The GITT results of LCO and DO-LCO. The figure includes the calculated diffusion coefficient. (b) Overpotential obtained from GITT results for LCO and DO-LCO.

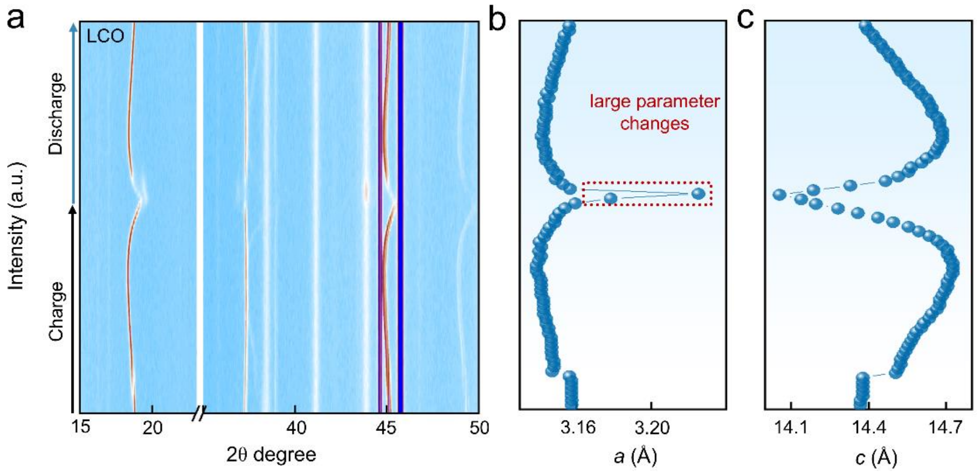

Figure S22. The *in-situ* XRD results of LCO during the initial charge and discharge processes and changes in cell parameters *a* and *c* obtained by refinement.

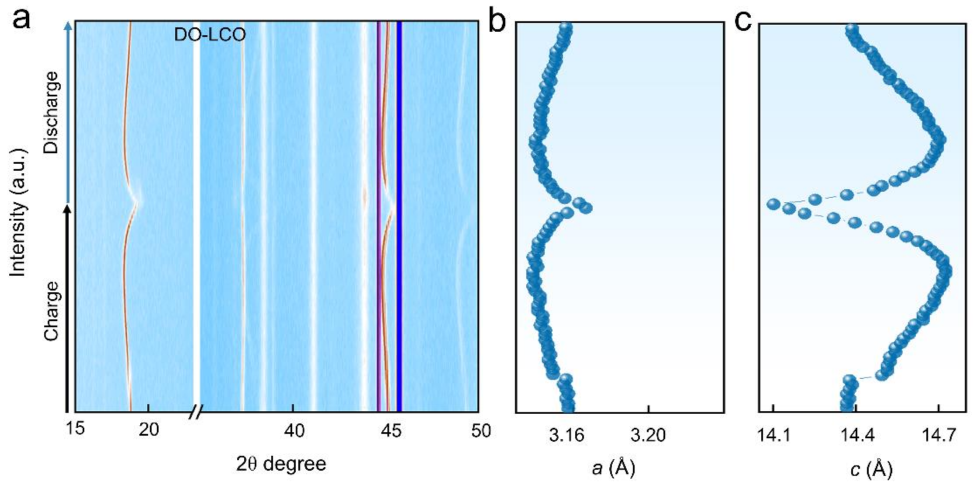

Figure S23. The *in-situ* XRD results of DO-LCO during the initial charge and discharge processes and changes in cell parameters *a* and *c* obtained by refinement.

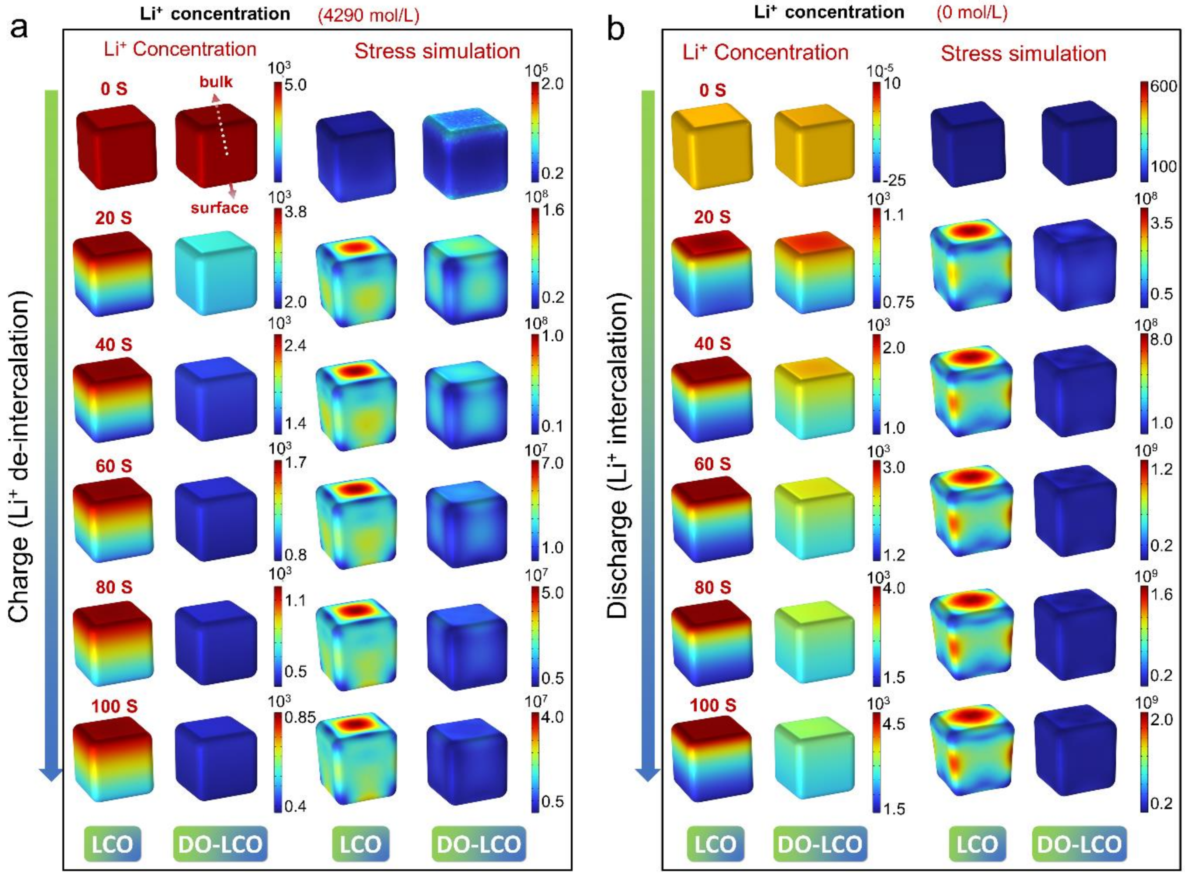

Figure S24. The simulation diagram of Li^+ concentration distribution and structure stress simulation diagram for LCO and DO-LCO at the bulk and surface with COMSOL software at 100 cycles.

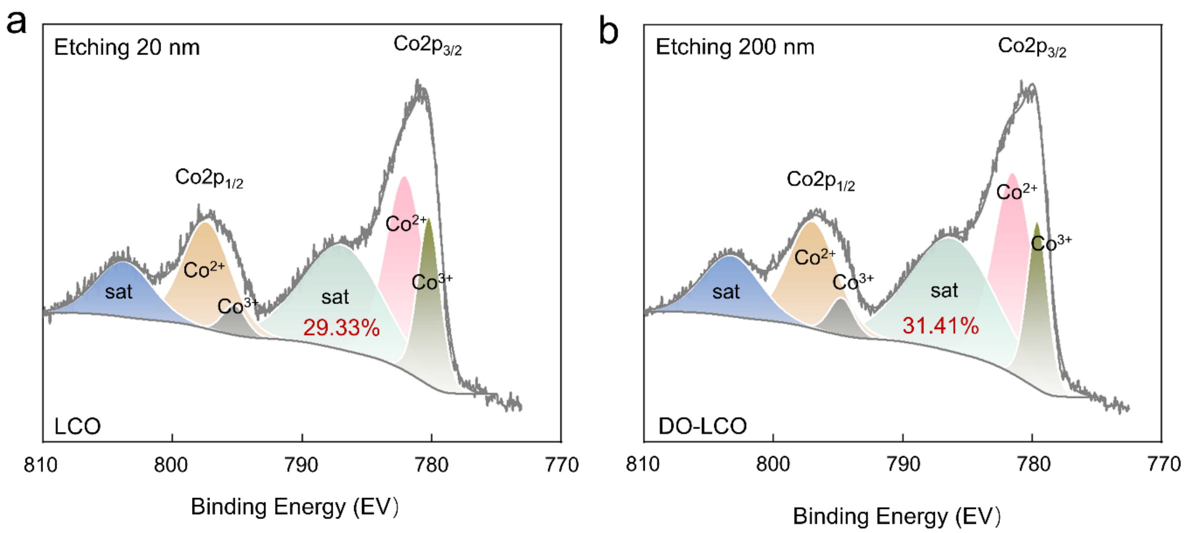

Figure S25. The Co 2p XPS for LCO (a) and DO-LCO (b).

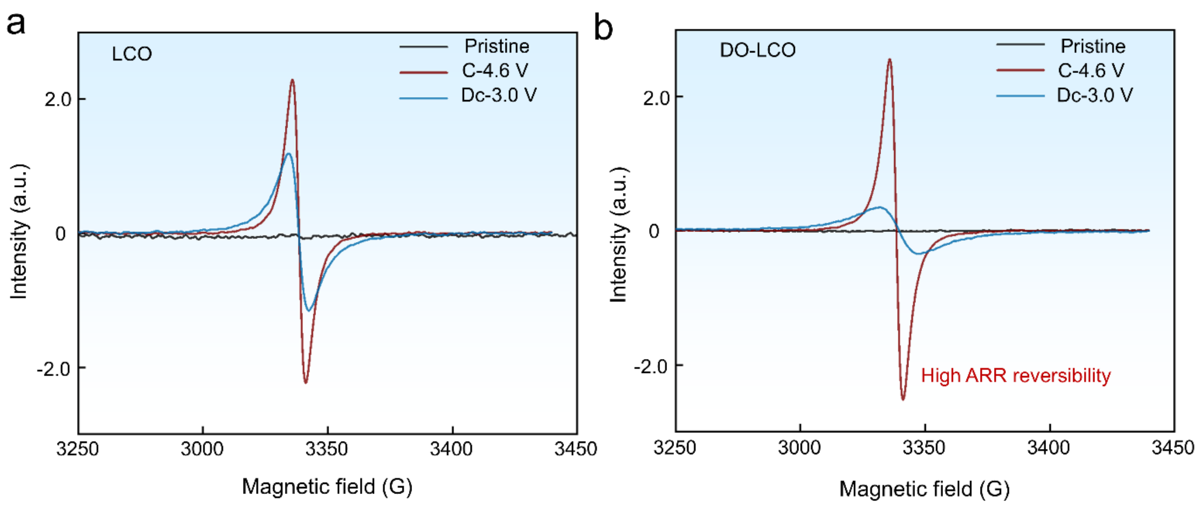

Figure S26. Comparison of *ex-situ* EPR results of LCO (a) and DO-LCO (b) at different states.

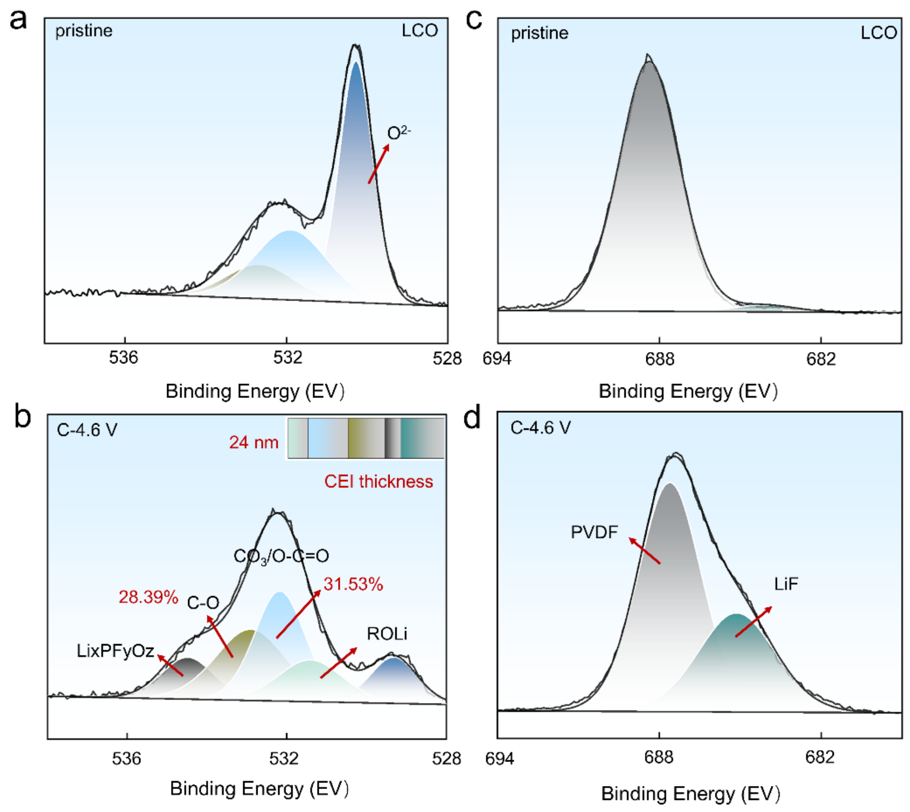

Figure S27. The fitted O 1s (a, b) and F 1s (c, d) XPS patterns for LCO at pristine and 4.6 V-charged states, the insert lateral histogram shows the calculated CEI thickness and components.

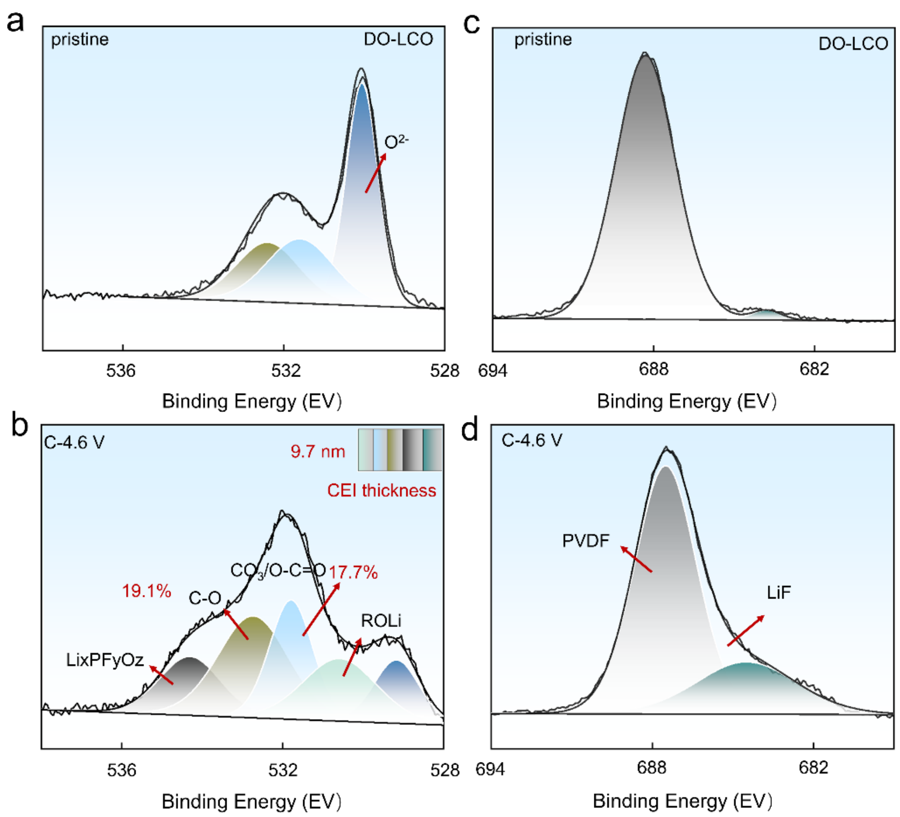

Figure S28. The fitted O 1s (a, b) and F 1s (c, d) XPS patterns for DO-LCO at pristine and 4.6 V-charged states, the insert lateral histogram shows the calculated CEI thickness and components.

According to the content of lattice oxygen, the thickness of cathode electrolyte interphase (CEI) is calculated by the formula $D=\ln(I_a/I_b)\lambda_{ads}\cos\theta_e t_{factor}$. Where I_a/I_b represents the ratio of the pristine/soaked lattice oxygen atom percentage; λ_{ads} represents the average free path of photoelectrons passing through the whole CEI film at the lattice oxygen peak, which is 2.84 nm; θ_e is the emission angle and the value is 0; t_{factor} refers to the correction factor of surface facial mask on spherical particles with a value of 0.67. Casa XPS software was used for fitting and quantitative analysis of the test data. The spectral data energy correction is to correct the peak potential energy value corresponding to C-C bond (conductive additive) in the C 1*s* spectrum to 284.8 eV. Based on the O 1*s* and F 1*s* XPS spectra, the ratio of different components in the original/soaked electrodes was obtained by the area of fitting. To more intuitively compare the CEI content of the pristine and soaked electrodes, and exclude the influence caused by the difference of the original electrode surface state, we carried out a quantitative analysis of each peak. Then, we obtained the relative percentage of CEI

components in the state of pristine and charge to 4.6 V from O 1*s* and F 1*s* of the *ex-situ* XPS spectra of LCO and DO-LCO cathodes. Thus, we can get the percentage of original/soaked lattice oxygen atoms.

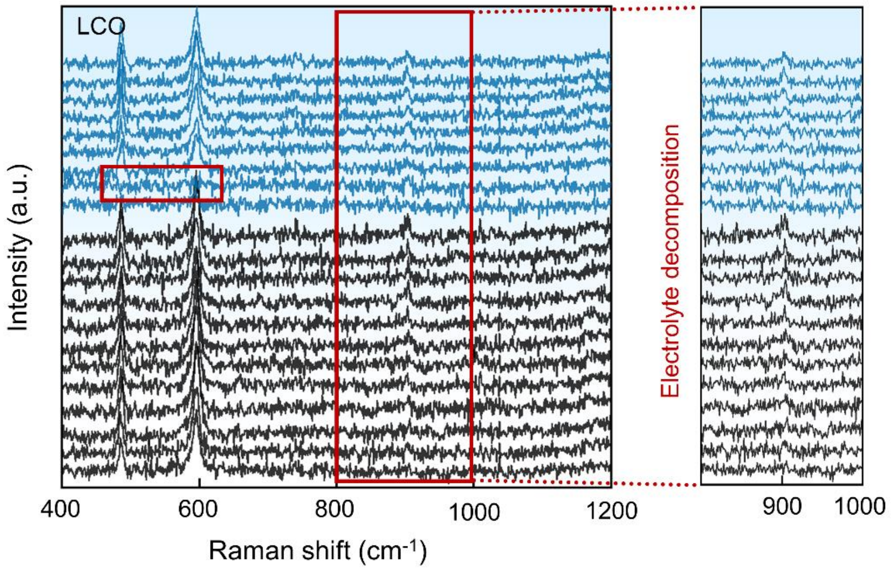

Figure S29. The *in-situ* Raman results of LCO during the initial charge and discharge processes and selected *in-situ* Raman data from the 800-1000 cm^{-1}.

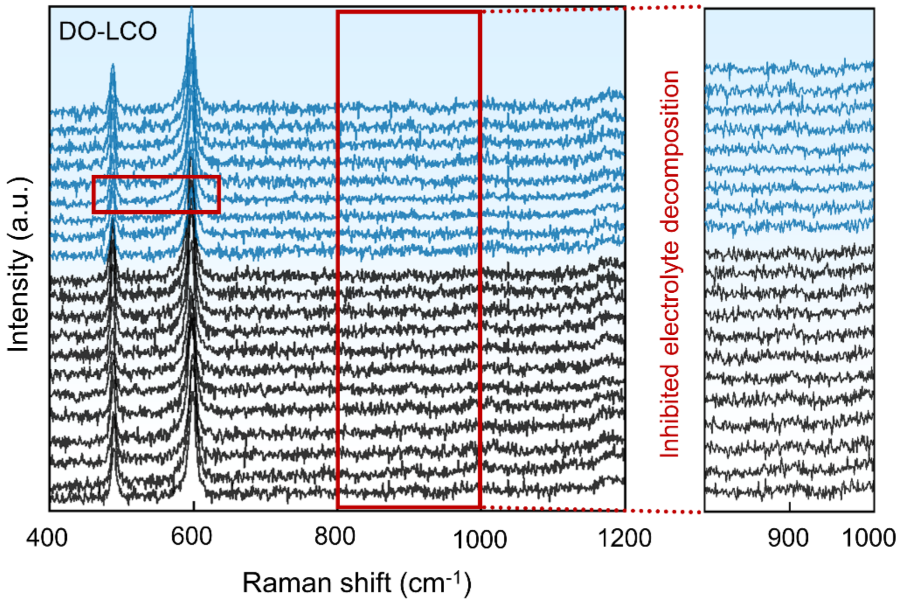

Figure S30. The *in-situ* Raman results of DO-LCO during the initial charge and discharge processes and selected *in-situ* Raman data from the 800-1000 cm^{-1}.

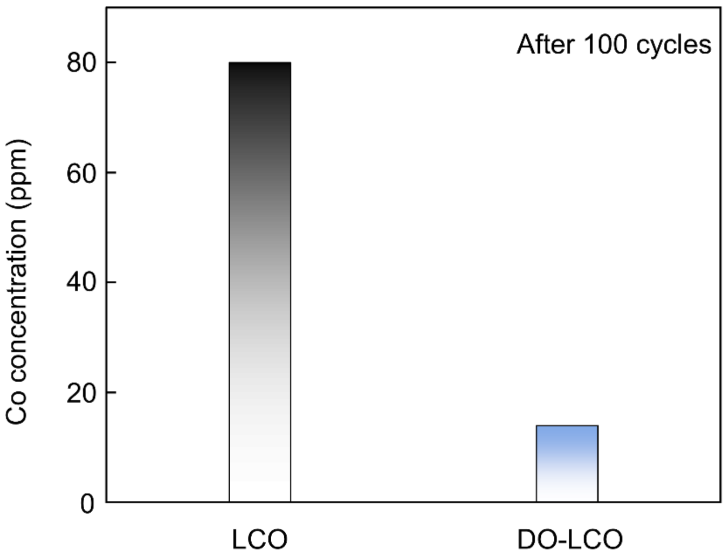

Figure S31. Contents of transition metal Co ions in electrolytes for LCO and DO-LCO materials after 100 cycles.

The dissolved Co contents in electrolyte after cycles were detected using ICP spectrometer. Both electrolytes were collected after the LCO and DO-LCO cathodes are charged/discharged 100 cycles. The Co content is 80 ppm in electrolyte for LCO, while only 15 ppm of the Co content is in electrolyte for DO-LCO, further proving that the diffusion optimization of Li^+ effectively inhibits the dissolution of Co (Figure S31).

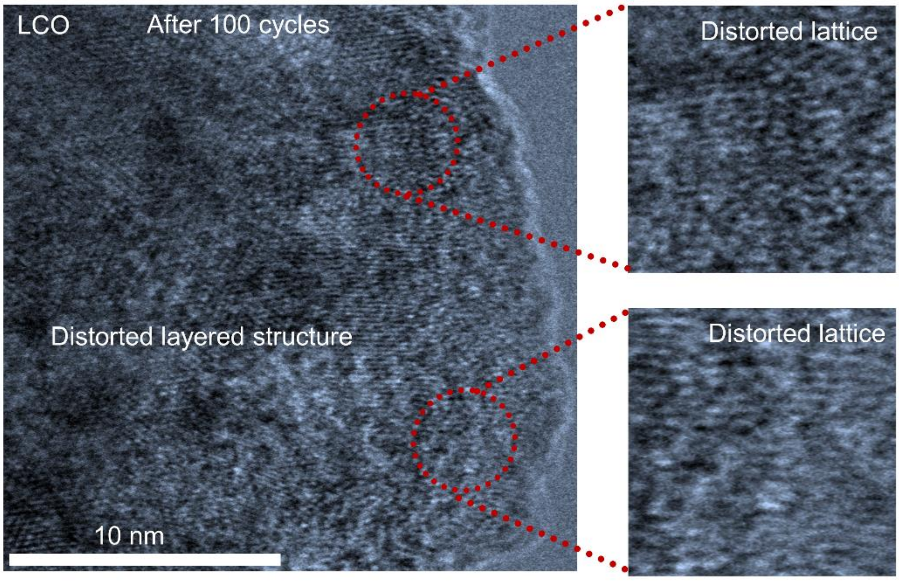

Figure S32. HRTEM data for LCO electrode collected after 100 cycles, the twisted lattice is highlighted by red circle.

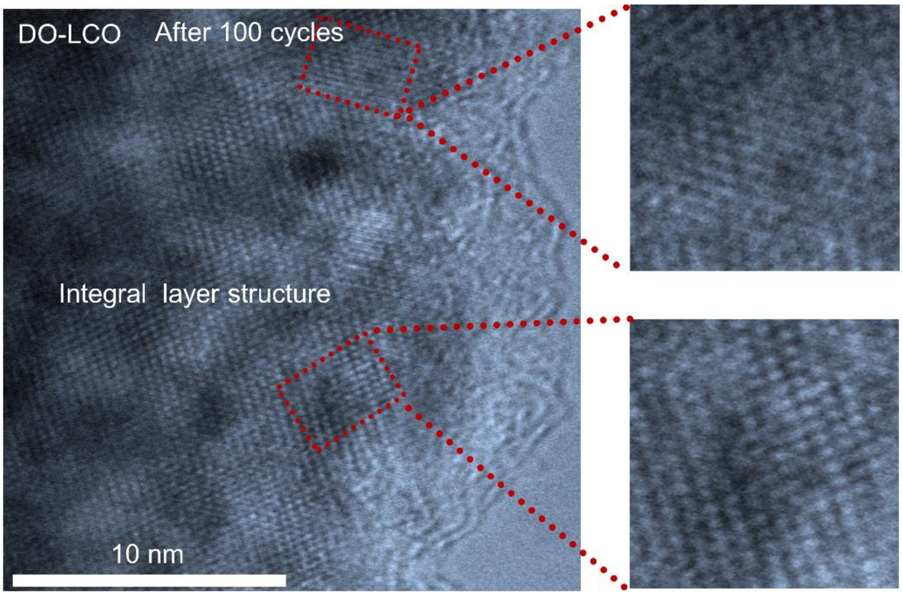

Figure S33. HRTEM data for DO-LCO electrode collected after 100 cycles, the clear lattice is highlighted by red rectangle.

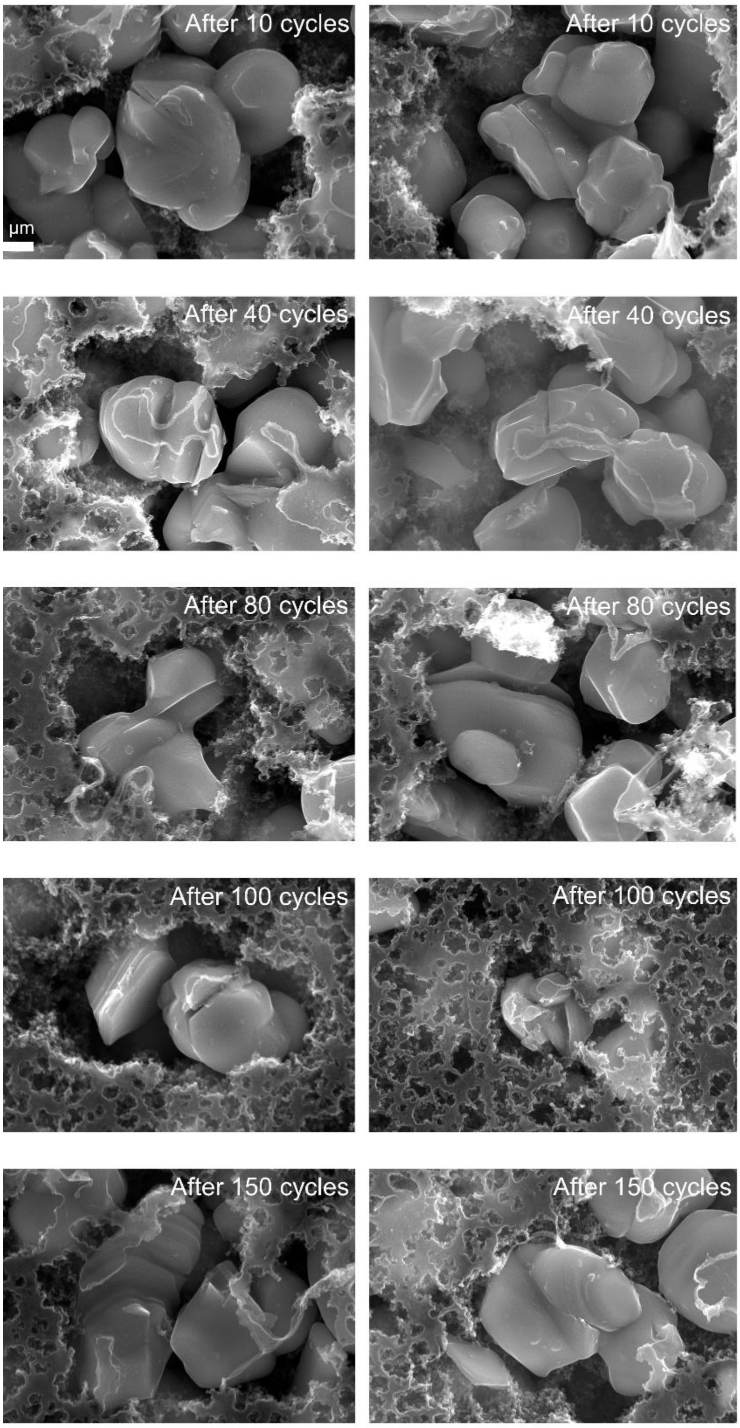

Figure S34. The SEM morphology of LCO material with different number of cycles.

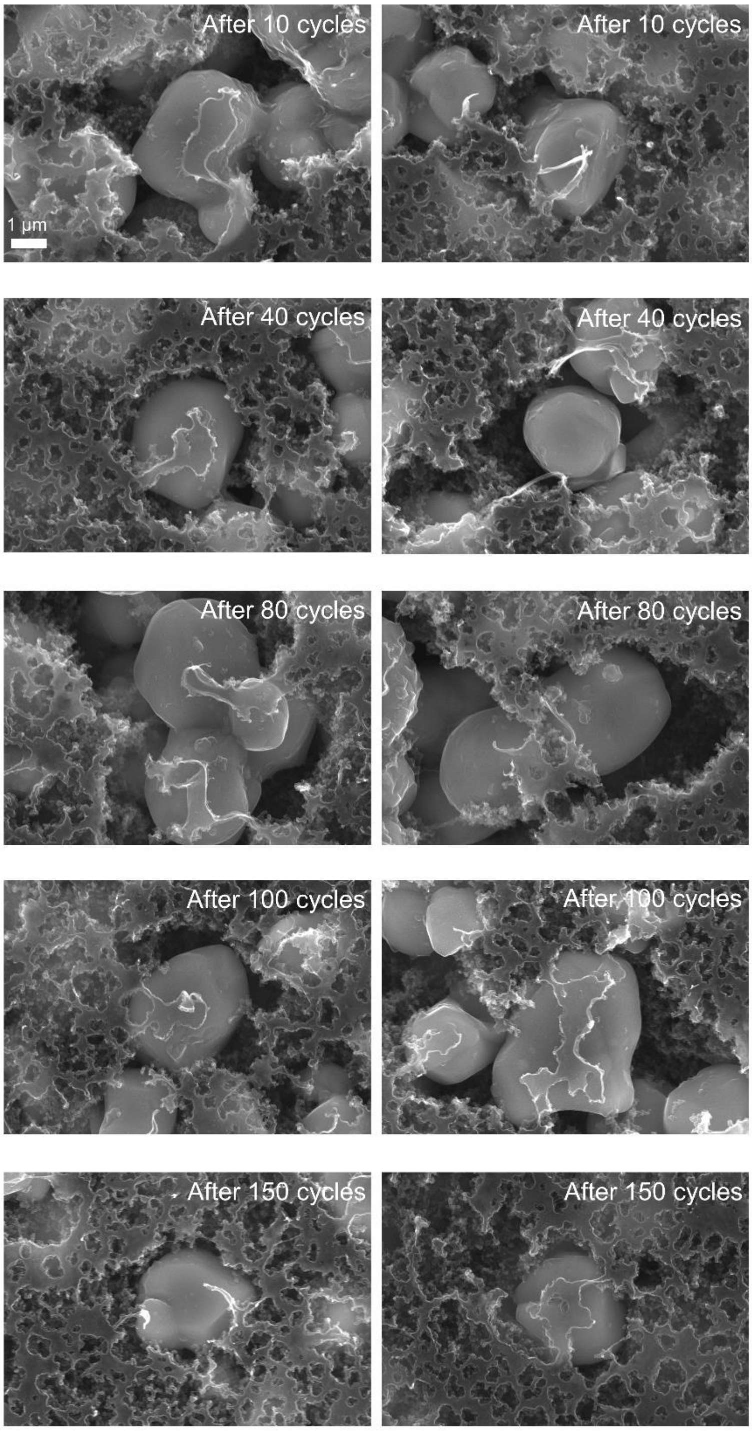

Figure S35. The SEM morphology of DO-LCO material with different number of cycles.

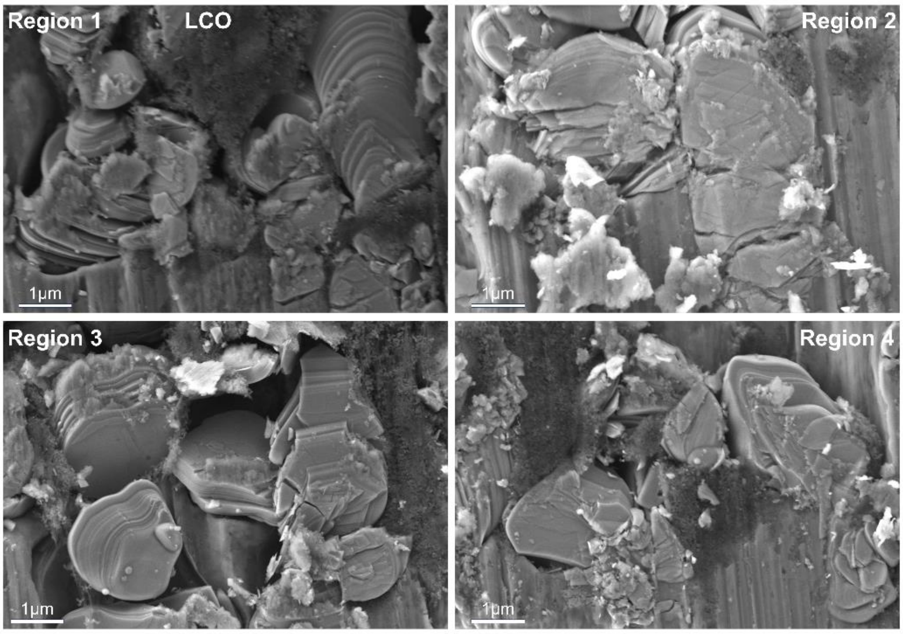

Figure S36. The cross-sectional SEM images of LCO material after 100 cycles.

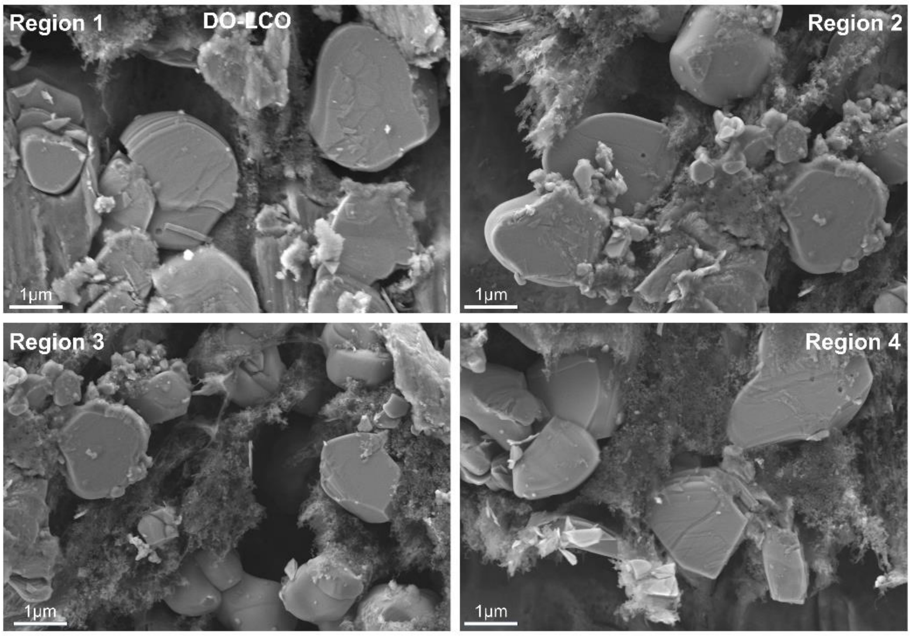

Figure S37. The cross-sectional SEM images of DO-LCO material after 100 cycles.

Table S1. ICP results (molar percentage content) of pristine and LCO, 0.3% DO-LCO, 0.5% DO-LCO and 0.7% DO-LCO samples.

	Li	Co	Ga	Ti	P
LCO	1	1			
0.3% DO-LCO	0.99	1	0.0015	0.0048	0.0096
0.5% DO-LCO	0.98	1	0.0024	0.0077	0.0160
0.7% DO-LCO	1	1	0.0037	0.0107	0.0213

Table S2. The refined crystal structure parameters of LCO sample using X-ray diffraction.

LCO (space group: R-3m) R_w=8.2 %, a=b=2.8138 (4) Å, c=4.0504 (5) Å					
Atom	site	x	y	z	Occ.
Li	*3a*	0	0	0	1
Co	3*b*	0	0	0.5	1
O	6*c*	0	0	0.2357 (9)	1

Table S3. The refined crystal structure parameters of 0.3% DO-LCO sample using X-ray diffraction.

0.3% DO-LCO (space group: R-3m) R_w=7.5 %, a=b=2.8145 (8) Å, c=4.0550 (3) Å					
Atom	site	x	y	z	Occ.
Li	*3a*	0	0	0	1
Co	3*b*	0	0	0.5	0.993 (4)
Ga	3*b*	0	0	0.5	0.003 (2)
Ti	3*b*	0	0	0.5	0.004 (1)
O	6*c*	0	0	0.2389 (9)	1

Table S4. The refined crystal structure parameters of 0.5% DO-LCO sample using X-ray diffraction.

DO-LCO (space group: R-3m) R_w=7.6 %, a=b=2.8141 (2) Å, c=4.0539 (7) Å					
Atom	site	x	y	z	Occ.
Li	*3a*	0	0	0	1
Co	3*b*	0	0	0.5	0.987 (7)
Ga	3*b*	0	0	0.5	0.006 (1)
Ti	3*b*	0	0	0.5	0.007 (5)
O	6*c*	0	0	0.2404 (9)	1

Table S5. The refined crystal structure parameters of 0.7% DO-LCO sample using X-ray diffraction.

0.7% DO-LCO (space group: R-3m) R_w=7.2 %, a=b=2.8157 (4) Å, c=4.0574 (3) Å					
Atom	site	x	y	z	Occ.
Li	*3a*	0	0	0	1
Co	3*b*	0	0	0.5	0.985 (6)
Ga	3*b*	0	0	0.5	0.006 (2)
Ti	3*b*	0	0	0.5	0.009 (7)
O	6*c*	0	0	0.2395 (9)	1

Table S6. Satellite Relative Area (%) of the Main Peak from Co 2p3/2 Spectra for LCO and LGTP-LCO at different etching depth.

% sat. area/ Etching depth	LCO	LGTP-LCO
	29.33 (20 nm)	31.41 (200 nm)
	30.01 (70 nm)	32.31 (250 nm)
	30.41 (120 nm)	32.46 (300 nm)
	31.18 (420 nm)	32.51 (600 nm)
	31.23 (720 nm)	32.51 (900 nm)
	32.23 (1020 nm)	32.56 (1200 nm)
	32.43 (1320 nm)	32.67 (1500 nm)
	34.20 (1720 nm)	32.95 (1900 nm)
	35.56 (2020 nm)	33.73 (2200 nm)

Table S7. Comparison of various electrochemical properties (initial capacity and capacity retention) of LCO at different current densities for hundreds of cycles in literatures.

Cycles	I (mA g^{-1})	Initial capacity (mA h g^{-1})	Capacity Retention (%)	Ref.
100	274 (1C)	196	94.6	[S5]
100	270 (1C)	180	84	[S6]
100	270 (1C)	165	84	[S7]
100	137 (0.5C)	200	90	[S8]
100	137 (0.5C)	208	88	[S9]
100	137 (0.5C)	185	93	[S10]
100	137 (0.5C)	202	86	[S11]
100	274 (1C)	211	92	this work
200	274(1C)	200	90	[S12]
200	274 (1C)	202	90	[S13]
200	274 (1C)	200	90	[S14]
200	274 (1C)	211	88	this work
300	274 (1C)	200	81.3	[S15]
300	274 (1C)	211	86	this work
400	274 (1C)	200	84	[S12]
400	274 (1C)	211	84	this work
500	200 (1C)	202	79.8	[S16]
500	185 (1C)	184	65.4	[S17]
500	274 (1C)	211	81	this work
600	274 (1C)	211	80	this work

Table S8. The angle and peak intensities corresponding to the H1-3 phase of LCO and DO-LCO.

Samples	LCO	DO-LCO
	18.29177/6156	18.29177/5813
	18.3049/6202	18.3049/5819
	18.31803/6116	18.3049/5709
	18.31803/6281	18.33116/5669
	18.34429/6233	18.35742/5818
	18.37056/6234	18.38369/5810
Angle/	18.40995/6202	18.42308/5789
Peak Intensities	18.46247/6190	18.4756/5870
	18.51499/5982	18.54125/5829
	18.59377/5947	18.62003/5620
	18.67255/5593	18.72507/5431
	18.77759/5356	18.83012/5085
	18.88264/5119	18.97455/4779
	19.00081/4797	19.05333/4593
	19.07959/4458	19.13211/4316
	19.19776/4103	-----

Table S9. The Atomic Percentages (%) of each component in the state of pristine and charge to 4.6 V from O 1s and F 1s XPS spectra of LCO and DO-LCO cathodes.

			pristine		C-4.6 V	
	Peak	Assignments	BE(eV)	At%	BE(eV)	At%
LCO	O1s	Lattice O	530.28	52.22	529.32	12.30
		ROLi	--	--	531.41	15.31
		CO_3/ O-C=O	531.90	32.82	532.18	31.53
		C-O	532.67	14.96	532.9	28.39
		$Li_xPF_yO_z$	--	--	534.48	12.47
	F1s	LiF	685.39	2.70	686.09	34.59
DO-LCO	O1s	Lattice O	530.06	46.56	529.32	18.07
		ROLi	--	--	531.25	19.34
		CO_3/ O-C=O	531.60	28.69	531.78	17.77
		C-O	532.40	24.75	532.42	19.06
		$Li_xPF_yO_z$	--	--	533.96	25.76
	F1s	LiF	685.20	1.87	685.66	25.43

Reference

[1] a) X. Deng, Z. Wei, C. Cui, Q. Liu, C. Wang, J. Ma, *J. Mater. Chem. A*, **2018**, *6*(9), 4013-4022; b) H. Liu, J. Xia, N. Zhang, H. Cheng, W. Bi, X. Zu, W. Chu, H. Wu, C. Wu, Y. Xie, *Nat. Catal.* **2021**, *4*, 342.

[2] a) K. Hayamizu, S. Seki, *Phys. Chem. Chem. Phys.* **2017**, *19*, 23483; b) C. Huang, F. Wang, S. Huang, J. Hong, S. Yuan, S. Hou, H. Jin, *J. Mater. Chem. A*, **2023**, *11*, 12034; c) N. Boaretto, P. Ghorbanzade, H. P. Furundarena, L. Meabe, J. M. L. Amo, I. E. Gunathilaka, M. Forsyth, J. Schuhmacher, A. Roters, S. Krachkovskiy, A. Guerfi, M. Armand, M. M. Ibañez, *Small* **2023**, 2305769.

[3] a) Y. Wang, Q. Zhang, Z. Xue, L. Yang, J. Wang, F. Meng, Q. Li, H. Pan, J. Zhang, Z. Jiang, W. Yang, X. Yu, L. Gu, H Li, *Adv. Energy Mater.* **2020**, *10*, 2001413; b) X. Zhu, T. U. Schülli, X. Yang, T. Lin, Y. Hu, N. Cheng, H. Fujii, K. Ozawa, B. Cowie, Q. Gu, S. Zhou, Z. Cheng, Y. Du, L. Wang, *Nat. Commun*. **2022**,*13*, 1565.

[4] L. Dahéron, R. Dedryvère, H. Martinez, M. Ménétrier, C. Denage, C. Delmas, D. Gonbeau, *Chem. Mater.* **2008**, *20*, 583–590.

[5] J. Chen, H. Chen, S. Zhang, A. Dai, T. Li, Y. Mei, L. Ni, X. Gao, W. Deng, L. Yu, et al. *Adv. Mater.* **2022**, *34* (42), 202204845.

[6] Y. Huang, Y. Zhu, H. Fu, M. Ou, C. Hu, S. Yu, Z. Hu, C. T. Chen, G. Jiang, H. Gu, et al. *Angew. Chem., Int. Ed.* **2021**, *60* (9), 4682-4688.

[7] W. Kong, D. Wong, K. An, J. Zhang, Z. Chen, C. Schulz, Z. Xu, X. Liu, *Adv. Funct. Mater.* **2022**, *32* (31), 2202679.

[8] X. Yang, C. Wang, P. Yan, T. Jiao, J. Hao, Y. Jiang, F. Ren, W. Zhang, J. Zheng, Y. Cheng, et al. *Adv. Energy Mater.* **2022**, *12* (23), 2200197.

[9] Y. Wang, Q. Zhang, Z. C. Xue, L. Yang, J. Wang, F. Meng, Q. Li, H. Pan, J. N. Zhang, Z. Jiang, et al. *Adv. Energy Mater.* **2020**, *10* (28), 2001413.

[10] H. Huang, Z. Li, S. Gu, J. Bian, Y. Li, J. Chen, K. Liao, Q. Gan, Y. Wang, S. Wu, et al. *Adv. Energy Mater.* **2021**, *11* (44), 2101864.

[11] J. Zhang, Q. Li, C. Ouyang, X. Yu, E. Hu, C. Ma, S. Li, R. Xiao, W. Yang, Y. Chu, Y. Liu, H. Yu, X. Yang, X. Huang, L. Chen, H. Li, *Nat. Energy* **2019**, *4*, 594–603.

[12] W. Zhang, X. Zhang, F. Cheng, M. Wang, J. Wan, Y. Li, J. Xu, Y. Liu, S. Sun, Y. Xu, et al. *J. Energy Chem.* **2023**, *76*, 557-565.

[13] A. Fu, Z. Zhang, J. Lina, Y. Zoua, C. Qinb, C Xua, P. Yan, K. Zhou, J Hao, X. Yang, Y. Cheng, D. Wu, Y Yang, M. Wang, J Zheng, *Energy Storage Mater.* **2022**, *46*, 406–416.

[14] W. Zhang, F. Cheng, M. Chang, Y. Xu, Y. Li, S. Sun, L. Wang, L. Xu, Q. Li, C. Fang, M. Wang, Y. Lu, J. Han, Y. Huang, *Nano Energy* **2023**, 2211-2855.

[15] K. Guo, C. Zhu, H. Wang, S. Qi, J. Huang, D. Wu, J. Ma, *Adv. Energy Mater.* **2023**, *13* (20), 2204272.

[16] M. Cai, Y. Dong, M. Xie, W. Dong, C. Dong, P. Dai, H. Zhang, X. Wang, X. Sun, S. Zhang, et al. *Nat. Energy* **2023**, *8* (2), 159-168.

[17] T. Cheng, Z. Ma, R. Qian, Y. Wang, Q. Cheng, Y. Lyu, A. Nie, B. Guo, *Adv. Funct. Mater.* **2021**, *31*, 2001974.
